# Regulation of p53 expression and apoptosis by vault RNA2-1-5p in cervical cancer cells

**DOI:** 10.18632/oncotarget.4948

**Published:** 2015-07-22

**Authors:** Lu Kong, Qi Hao, Ying Wang, Ping Zhou, Binbin Zou, Yu-xiang Zhang

**Affiliations:** ^1^ Department of Biochemistry and Molecular Biology, School of Basic Medical Sciences, Capital Medical University, Beijing, China; ^2^ Department of Bioinformatics and Computer Science, School of Biomedical Engineering, Capital Medical University, Beijing, China; ^3^ Cancer Institute of Capital Medical University, Beijing, China; ^4^ Beijing Key Laboratory for Cancer Invasion and Metastasis Research, Capital Medical University, Beijing, China

**Keywords:** non-coding microRNAs, p53, cervical cancer, VTRNA2-1-5p, apoptosis

## Abstract

nc886 or VRNA2-1 has recently been identified as a noncoding RNA instead of a vault RNA or a pre-microRNA. Several studies have reported that pre-miR-886 plays a tumor-suppressive role in a wide range of cancer cells through its activity as a cellular protein kinase RNA-activated (PKR) ligand and repressor. However, by sequencing stem-PCR products, we found that a microRNA originating from this precursor, vault RNA2-1-5p (VTRNA2-1-5p), occurs in cervical cancer cells. The expression levels of the predicted targets of VTRNA2-1-5p are negatively correlated with VTRNA2-1-5p levels by quantitative reversion transcription PCR (qRT-PCR). Previous results have shown that VTRNA2-1-5p is overexpressed in human cervical squamous cell carcinomas (CSCCs) compared with adjacent healthy tissues. Inhibition of VTRNA2-1-5p increases Bax protein expression and apoptotic cell death in cervical cancer cells. Our findings suggest that VTRNA2-1-5p has oncogenic activity related to the progression of cervical cancer. Here, we report that VTRNA2-1-5p directly targeted p53 expression and functioned as an oncomir in cervical cancer. VTRNA2-1-5p inhibition decreased cervical cancer cell invasion, proliferation, and tumorigenicity while increasing apoptosis and p53 expression. Interestingly, VTRNA2-1-5p inhibition also increased cisplatin-induced apoptosis of HeLa and SiHa cells. In human clinical cervical cancer specimens, low p53 expression and high VTRNA2-1-5p expression were positively associated. In addition, VTRNA2-1-5p was found to directly target the 5′ and 3′ untranslated regions (UTRs) of p53. We propose that VTRNA2-1-5p is a direct regulator of p53 and suggest that it plays an essential role in the apoptosis and proliferation of cervical cancer cells.

## INTRODUCTION

Cervical cancer is the second-most common cancer in women, and the integration of human papillomavirus (HPV) DNA into the host genome is a typical, although not exclusive, step in cervical carcinogenesis [1, 2]. MicroRNAs have been suggested to play an important role in cervical carcinogenesis [3–5]. In a previous investigation of miRNAs that are differentially expressed in cervical squamous cell carcinoma (CSCC) and adjacent healthy tissue, we found that miR-886-5p is highly overexpressed in cancerous tissue. Further research showed that miR-886-5p inhibits the apoptosis of cervical cancer cells by down-regulating the expression of Bax [6].

Pre-miR-886 is composed of approximately 102 nucleotides and located on chromosome 5q31.1, and it has been considered a miRNA precursor because its mature forms, miR-886-5p and miR-886-3p, which can be captured by high-throughput sequencing, might be produced by Dicer [7, 8]. However, experimental evidence suggests that miR-886 is not a canonical miRNA but a vault RNA (VTRNA) because the sequence alignments clearly identify this sequence as a VTRNA homolog or a molecule associated with the vault complex [9, 10]. Hence, the revised name “VTRNA2-1” has been approved by the HUGO Gene Nomenclature Committee (http://www.genenames.org/) and included in various databases, such as the NCBI (http://www.ncbi.nlm.nih.gov/) and the UCSC genome browser (http://genome.ucsc.edu/). Recently, Lee *et al*. provided solid experimental evidence that pre-miR-886 is neither a VTRNA nor a canonical miRNA, although they could not rule out the possibility that only a minute amount of pre-miR-886 is associated with major vault protein (MVP) and has a biological function [11]. Despite the divergence in the characterization of pre-miR-886, all the available data are consistent in suggesting that human VTRNAs are RNA polymerase III (Pol III) transcripts with defective stem-loop secondary structures.

To date, human studies have identified four VTRNA genes clustered on chromosome 5 (VTRNA1-1, 1-2, 1-3 and 2-1) and a pseudogene on chromosome X [10]. VTRNA associates with the vault complex, a large hollow barrel-shaped RNP complex with a size of 13 MDa. The vault consists of multiple components, including MVP, which constitutes over 70% of the vault complex; two minor proteins, TEP1 and VPARP; and VTRNAs. VTRNAs only represent approximately 5% of the particle mass, and approximately 20% of the VTRNAs are associated with vaults, with the majority localized in the soluble fraction. We can’t rule out miR-886 functional role as a VTRNA despite the fact that VTRNA2-1 was not detected in the P100 fraction; instead, 97% of VTRNA2-1 is found in the S100 fraction when pre-miR-886 is co-fractionated with MVP.

Thus, additional work is required to elucidate the function of VTRNA2-1 and its interaction with vault in the future. The vault complex may play a role in cellular resistance to anti-cancer drugs [12]. Pre-miR-886 is repressed in a wide range of cancer cells, and Lee *et al*. have suggested a tumor-suppressive role for pre-miR-886; however, the exact nature of its role remains unclear.

Taking our previous work into consideration, we suggest that miR-886 is a VTRNA. We cannot rule out that it may have vault-independent functions, such as that of a microRNA precursor. Generally, small RNAs regulate the expression of their targets at the post-transcriptional or translational level by their incorporation into the RNA-induced silencing complex (RISC) [13]. The Argonaute (AGO) protein is an important protein component of RISC. Thus, the isolation of AGO-associated small RNAs and the target genes of miRNAs constitutes an important approach for identifying functional small RNAs and their target genes [14–16].

In the present study, we investigated the effect of VTRNA2-1-5p inhibition on cervical cancer cells and found that such inhibition resulted in cell apoptosis and p53 up-regulation. Also known as protein 53 or tumor protein 53, p53 is a tumor suppressor protein with a key upstream role in the apoptosis pathway in cancer [17]. In fact, the MVP promoter region may contain a functional p53-binding site (unpublished results of Berger *et al*) [12]. Furthermore, in chromatin immunoprecipitation (ChIP) experiments, Park *et al*. observed that p53 binds directly to the MVP promoter, and they suggested that p53 may act as a major transcriptional factor for the MVP gene [18]. Because we can demonstrate that p53 is a direct target of VTRNA2-1-5p, we suggest that miR-886 is a VTRNA.

## RESULTS

### Does VTRNA2-1-5p occur in cervical tissues and cells?

**Figure 1:**
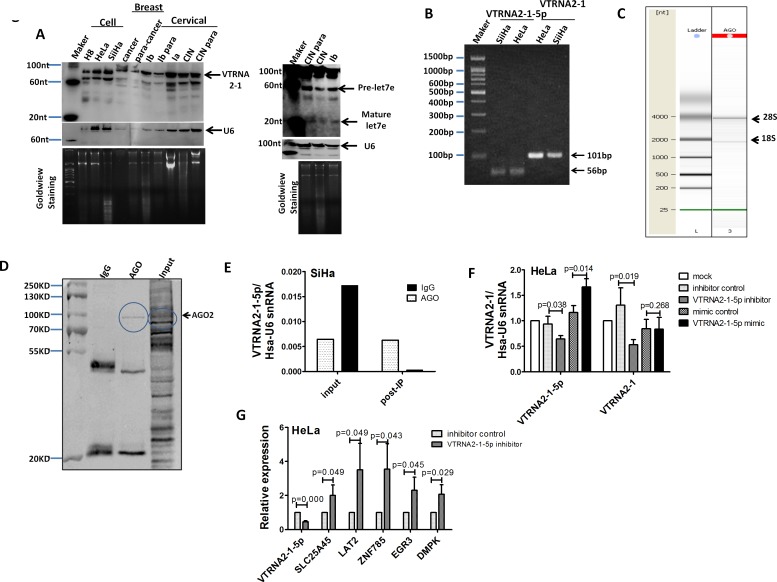
VTRNA2-1-5p presence in cervical tissues and cells **A.** Northern blot showing VTRNA2-1 in three cervical cell lines: one breast tumor specimen (cancer) and the surrounding normal tissue control (para-cancer); one hyperplastic cervical squamous epithelium (CINIII) and the surrounding normal tissue control (para-CINIII); and one different stage cervical cancer tissue (Ia, Ib stage) and the surrounding normal tissue control (Ib para) from the same patient. The blot was evaluated with a high-sensitivity probe specific to VTRNA2-1-5p. Human U6 was used as a loading control. Molecular size in nucleotides is indicated on the left. To rule out technical artifacts, let-7e was detected as a control in cervical tissues using a high-sensitivity probe specific to let-7e. **B.** After stem-loop qPCR of VTRNA2-1-5p and qRT-PCR of VTRNA2 in HeLa and SiHa, the products were resolved on a 2% agarose gel and visualized using GoldView staining. The bands consisted of the expected 102-nt band of VTRNA2 and a 54-nt band representing VTRNA2-1-5p. (C, D, E) VTRNA2-1-5p was produced through the mechanism of RISC. **C.** Quality check of RNAs isolated from anti-AGO-immunoprecipitated RNPs performed using a Bioanalyzer 2100 system prior to sequencing. **D.** Detection of AGO2 expression by Western blotting in AGO2 RNP complexes immunoprecipitated with anti-AGO2 or mouse IgG; the input samples are shown. **E.** Levels of VTRNA2-1-5p from RNA complex and input samples were detected with stem-loop qRT-PCR. **F.** Suppression of VTRNA2-1-5p in HeLa cells decreased the level of VTRNA2. **G.** qRT-PCR detection of mRNA expression of the predicted targets under suppression of VTRNA2-1-5p in HeLa. The results shown represent the mean±SD of at least 3 independent experiments. *p < 0.05, **p < 0.01; two-tailed Student’s *t*-test.

In 2011, the Lee laboratory failed to detect miR-886-5p and miR-886-3p in various human organs and certain cancer cells by Northern blotting [11, 17]. This result raised the question of whether VTRNA2-1-5p is present in cervical cancer tissues or cells. To address this question, we measured VTRNA2-1-5p levels by Northern blotting using a highly sensitive probe specific to VTRNA2-1-5p. The expected band of ~22 nt was not observed in cervical or breast tissues or in cervical cells, whereas mature let-7e was detected in cervical cancer tissues (Figure 1A). Our data are consistent with the report of Lee *et al*. However, we detected two bands of ~90 nt and ~70 nt in cervical and breast tissues and in cervical cancer cells. Because the intracellular levels of VTRNA2-1-5p may be too low for detection by Northern blotting, we electrophoresed and sequenced VTRNA2-1-5p stem-loop qPCR products and sequenced VTRNA2-1 from SiHa and HeLa cells as a control (Figure 1B). The alignment results of VTRNA2-1-5p and VTRNA2-1 showed that VTRNA2-1-5p is present in cells (Supplementary Figure S1). Next, we tested whether VTRNA2-1 is processed into mature miRNAs by the RISC complex. An RNP immunoprecipitation (RIP) assay was used, and endogenous AGO-containing miRNP complexes in SiHa cells and their associated mRNAs were pulled down using an anti-AGO 2A8 antibody. The migration of the anti-AGO RIP sample showed two main peaks at approximately 2000 and 4000 nucleotides, which corresponded to 18S and 28S ribosomal RNA, respectively (Figure 1C). The Western blotting results confirmed that AGO was specifically immunoprecipitated by the anti-AGO antibody but not by control mouse IgG (Figure 1D). The VTRNA2-1-5p in the RNP complex was also quantitated by stem-loop qPCR. The VTRNA2-1-5p content of the anti-AGO RIP RNA complex was 22-fold higher than that of the control mouse IgG RIP RNA complex (0.006 *vs*. 0.0003) (Figure 1E). Because VTRNA2-1-5p occurs in the cell and VTRNA2-1 can be processed into mature VTRNA2-1-5p, changes in the VTRNA2-1-5p level should impact the level of VTRNA2-1. Therefore, we performed qRT-PCR to determine the VTRNA2-1 level under VTRNA2-1-5p suppression and overexpression in HeLa cells. Consistent with our predictions, VTRNA2-1-5p inhibitors significantly down-regulated the level of VTRNA2-1 (*p* < 0.05), whereas exposure of the cells to VTRNA2-1-5p mimics did not impact the level of VTRNA2-1 (*p* > 0.05, Figure 1F), which was highly abundant (~10^5^ molecules per cell) in HeLa. Another important question is whether VTRNA2-1-5p was functional as a mature miRNA. In the reports of miranda [19], PITA and Lee *et al*. [11], five target genes of hsa-miR-886-5p were predicted, and we detected the expression of these genes in HeLa cells under VTRNA2-1-5p suppression by qRT-PCR (for primer information, refer to Supplementary Table S4). The results showed that when VTRNA2-1-5p was 2-fold down-regulated, the five predicted targets, including ZNF785, SLC25A45, LAT2, EGR3 and DMPK, were up-regulated by 3.5-fold, 2.0-fold, 3.5-fold, 2.3-fold and 2.1-fold, respectively (*n* = 3, *p* < 0.05, Figure 1G). Thus, we concluded that VTRNA2-1-5p may be a functional mature miRNA.

### Increased expression of VTRNA2-1-5p and reduced expression of p53 in cervical cancer tissues and cells

**Figure 2:**
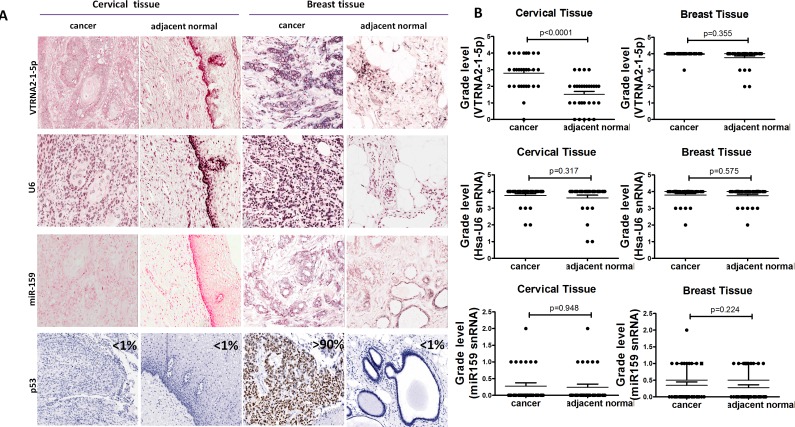
Comparison of VTRNA2-1-5p expression in cervical cancer tissue with inactivated p53 and in breast cancer tissue with mutated p53 **A.** 3′-DIG-labeled VTRNA2-1-5p LNA probe, snRNA U6 (positive control probe), miR-159 (negative control probe), and p53 antibodies were used. All the ISH images (×200) show intermediate to high staining intensity for VTRNA2-1-5p. Cervical cancer tissue showed more intense staining than adjacent normal tissue. Immunostaining for p53 was essentially negative in cervical tissue and normal breast tissue but strong in breast cancer tissue (×200). All the images were taken with an Aperio ImageScope system. **B.** Statistical graph showing VTRNA2-1-5p expression, human snRNA U6 expression and miR-159 expression in cervical and breast tissue. *n* = 31; nonparametric tests.

According to the graded ISH results, the expression of VTRNA2-1-5p was consistently higher in cervical cancer tissues (average grade = 2.79) than in adjacent normal tissues (average grade = 1.52, *p* < 0.0001, *n* = 31), whereas the expression of U6 in cervical cancer tissues (average grade = 3.75) was similar to that in normal tissues (average grade = 3.62, Figure 2A and 2B, Supplementary Figure S2).

Immunohistochemical staining for p53 was negative in the normal cervical tissue adjacent to tumors and in normal breast tissue (Figure 2A, left and right), which may have been caused by the low p53 levels in normal tissues. p53 staining was also low in cervical cancer tissue (Figure 2A, left). VTRNA2-1-5p was highly expressed in both breast cancer tissue and the adjacent normal breast tissue (average grade = 3.98 and 3.77, respectively, Figure 2A and 2B), whereas strong expression of p53 ( > 90%) was only observed in the breast cancer tissue (Figure 2A, Right). Thus, the levels of VTRNA2-1-5p and p53 expression differ in cervical and breast tissues.

**Figure 3:**
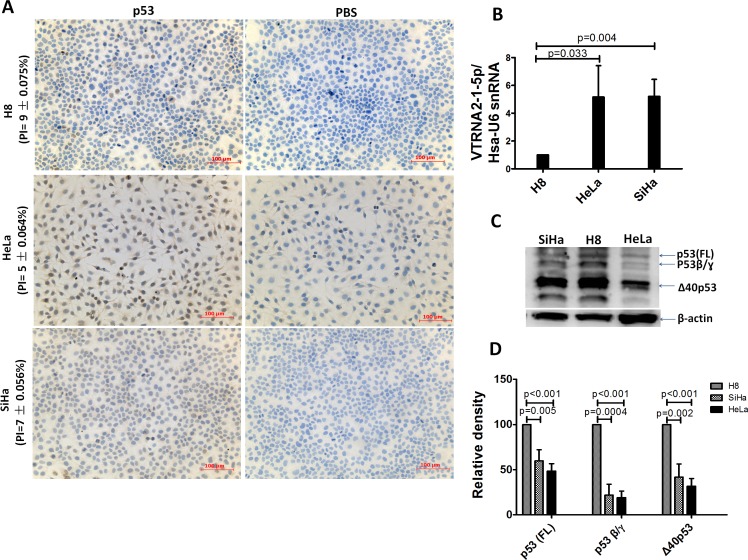
Comparison of VTRNA2-1-5p expression in three cervical cell lines with low p53 expression **A.** Low p53 expression assessed by IHC in H8, HeLa, and SiHa cell lines (*n* = 6, Supplementary Table 3). **B.** Overexpression of VTRNA2-1-5p in SiHa and HeLa cells; under-expression of VTRNA2-1-5p in H8 cells is shown by real-time stem-loop RT-PCR. The results represent the mean±SD of at least 3 independent experiments. **C.** Three wild-type p53 isoforms (FLp53, p53β and Δ40p53) were detected in the three cell lines by Western blotting. **D.** Statistical graph showing p53 isoform expression in three cell lines. Under identical levels of total protein (50 μg), p53 isoform expression in HeLa was lower than that in SiHa or H8 cells (*n* = 3).

The expression of p53 was also assessed by IHC and Western blotting in three cervical epithelial cell lines (H8, SiHa and HeLa). We first determined the VTRNA2-1-5p expression level in these cell lines, and the results showed that VTRNA2-1-5p was present at a higher level in HeLa and SiHa cells than in H8, which is an HPV 16-immortalized human cervical mucosal epithelial cell line (Figure 3B). The positive indexes for p53 were 9±0.075% (H8), 7±0.056% (SiHa), and 5±0.064% (HeLa) (Figure 3A and Supplementary Table S6). When the total amounts of protein were equivalent (i.e., 50 μg), the levels of the various p53 isoforms in the H8 cells were all higher than those found in the SiHa and HeLa cells (Figure 3C and 3D). Evidently, VTRNA2-1-5p is overexpressed in cervical cancer tissue and cell lines, whereas p53 is expressed at low levels.

### VTRNA2-1-5p promotes cervical cancer cell proliferation and invasion

**Figure 4:**
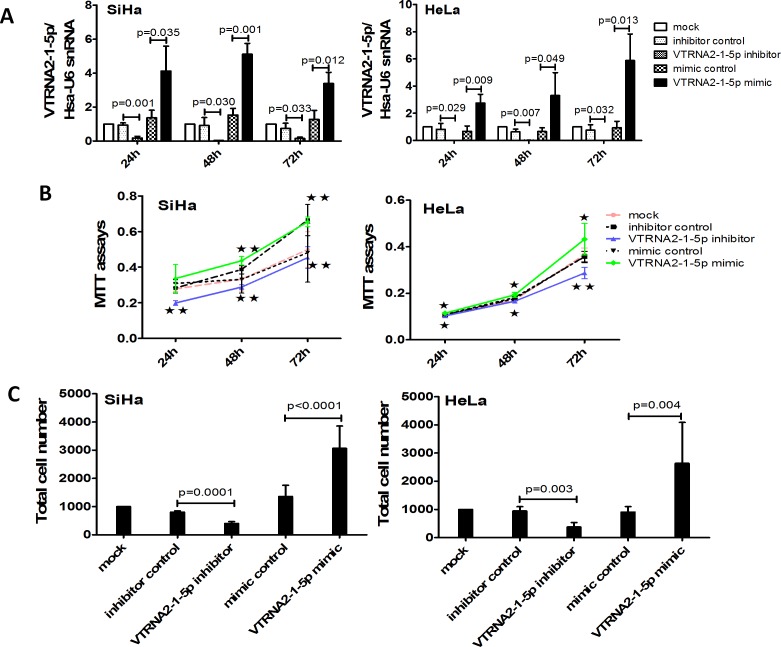
VTRNA2-1-5p knockdown decreases and overexpression enhances cervical cancer cell growth and invasion *in vitro* **A.** Transfection efficacy rates of HeLa and SiHa cells 24, 48 and 72 hours after transfection with 50 nmol/L of a VTRNA2-1-5p mimic or a negative control or after no transfection. **B.** Overexpression of VTRNA2-1-5p promotes SiHa and HeLa cell proliferation, whereas down-regulation of VTRNA2-1-5p inhibits SiHa and HeLa cell proliferation. **C.** VTRNA2-1-5p knockdown decreases SiHa and HeLa cell invasion, whereas overexpression increases SiHa and HeLa cell invasion. The data represent the mean±SD (*n* = 6). *p < 0.05, **p < 0.01; two-tailed Student’s *t*-test.

To measure cell proliferation and invasion, HeLa and SiHa cells were transfected with a VTRNA2-1-5p mimic and a VTRNA2-1-5p inhibitor as well as their respective negative controls. We then evaluated transfection efficiency by stem-loop qPCR at various time points, and the results showed that the VTRNA2-1-5p mimic significantly increased the levels of VTRNA2-1-5p at 24, 48 and 72 hours (*p* < 0.05), whereas the inhibitor decreased these levels (*p* < 0.01, *n* = 6) (Figure 4A).

The MTT assay results showed that cell proliferation was enhanced in HeLa and SiHa cells transfected with the VTRNA2-1-5p mimic (Figure 4B). Conversely, cell proliferation was reduced in cells transfected with the VTRNA2-1-5p inhibitor (Figure 4B). Less cell proliferation in case of HeLa cells compared to SiHa might be caused by the difference of cells growth. The growth speed in SiHa mock showed quicker than that of in HeLa mock. The results of the Transwell invasion assays showed that treatment with the VTRNA2-1-5p inhibitor for 24 hours reduced the invasive activity of SiHa and HeLa cells (SiHa with inhibitor: *p* = 0.0001; HeLa with inhibitor: *p* = 0.003; *n* = 6), whereas treatment with the mimic enhanced this activity (SiHa with mimic: *p* < 0.0001; HeLa with mimic: *p* = 0.004, *n* = 6; Figure 4C). We also evaluated transfection efficiency by stem-loop qPCR at 24 hours and found that the VTRNA2-1-5p mimic significantly increased the levels of VTRNA2-1-5p (*p* < 0.05, *n* = 3) at 24 hours (Supplementary Figure S3A). These results show that VTRNA2-1-5p promoted cervical cancer cell proliferation and invasion *in vitro*.

### VTRNA2-1-5p promotes tumor cell growth *in vitro*

**Figure 5:**
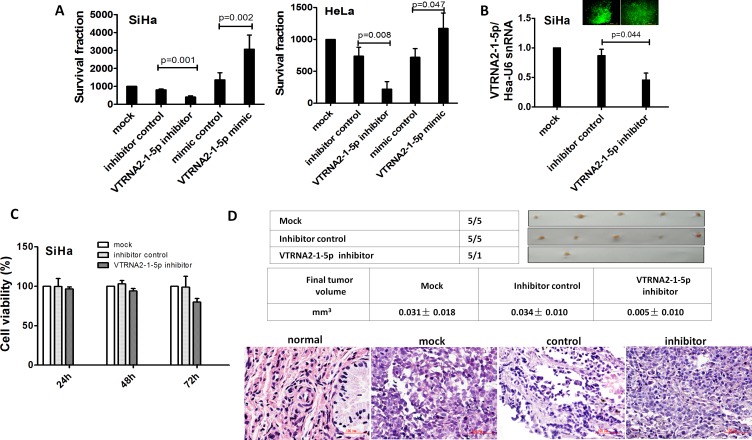
VTRNA2-1-5p promotes tumor cell growth *in vitro* and *in vivo* **A.** Colony formation assays were performed on SiHa and HeLa cell lines untreated or transfected with the negative control or the VTRNA2-1-5p mimic or inhibitor (*n* = 6). **B.** SiHa cells in 6-well plates were stably transfected with 1.0 μg VTRNA2-1-5p inhibitor GFP plasmid or negative control GFP tag vector, and inhibition was evaluated by real-time stem-loop PCR (*n* = 3). **C.** Stably transfection cells (*n* = 6) were examined for cell viability using the MTT assay as described in the Materials and Methods section. **D.** Xenografts in nude mice. Untreated, negative control vector transfected, and VTRNA2-1-5p-inhibitor transfected SiHa cells were s.c. injected into the posterior flanks of nude mice (*n* = 5). The graph shows data obtained from tumor tissues extracted from the various groups after 2 weeks. The tumor volumes were calculated, and the data are presented as the mean±SD. H&E staining was performed on mouse tumor tissue untreated or transfected with the VTRNA2-1-5p inhibitor or negative control vector. Magnification×200 (H&E). The two-tailed Student’s *t*-test was used.

To evaluate the growth of SiHa and HeLa cervical cancer cells transfected with a VTRNA2-1-5p mimic and inhibitor, colony formation by these cells was measured. The mimic-transfected GFP-positive cells formed larger colonies than the non-transfected controls or cells transfected with an empty vector (*p* = 0.002 for SiHa and *p* = 0.047 for HeLa cells, Figure 5A, Supplementary Figure S3B). Conversely, the inhibitor-transfected GFP-positive cells formed fewer and smaller colonies relative to the control cells (*p* = 0.001 for SiHa and *p* = 0.008 for HeLa, *n* = 6, Figure 5A, Supplementary Figure S3B). The transfection efficiency was verified by GFP expression. These data provide *in vitro* evidence for the growth-promoting role of VTRNA2-1-5p.

### VTRNA2-1-5p inhibitor decreases tumorigenicity *in vivo*

To confirm our findings, an *in vivo* model was employed. SiHa cell lines were stably transfected with a plasmid expressing a VTRNA2-1-5p inhibitor, and the knockdown efficiency of selected GFP-positive cells was evaluated by stem-loop qPCR (Figure 5B). VTRNA2-1-5p was reduced by nearly 50% in the cells expressing the VTRNA2-1-5p inhibitor (Figure 5B). Untreated SiHa cells and cells transfected with a control vector or with the plasmid expressing the VTRNA2-1-5p inhibitor were administered through subcutaneous (s.c.) injections into the posterior flank of nude mice. Two weeks after the injection, tumors developed in 100% of the untreated (mock) and control vector-transfected mice, whereas tumors developed in only 20% of the mice that had received VTRNA2-1-5p inhibitor-expressing cells (Figure 5C, upper). Moreover, the average tumor volume in the VTRNA2-1-5p inhibitor group (0.005±0.010 mm^3^) was smaller than the average tumor volume in the untreated (0.031±0.018 mm^3^, *p* = 0.03) and control vector-transfected animals (0.034±0.010 mm^3^, *p* = 0.001, *n* = 5, Figure 5C, middle). We also performed H&E staining of tumors from the mock, vector control-, and inhibitor-transfected SiHa cells (Figure 5C, lower). H&E staining of the tumors showed that all were CSCCs. Overall, our findings suggest that introducing a VTRNA2-1-5p inhibitor may serve to limit the growth of cervical cancer cells.

### VTRNA2-1-5p inhibition enhances cisplatin-induced apoptosis of cervical cancer cells

**Figure 6:**
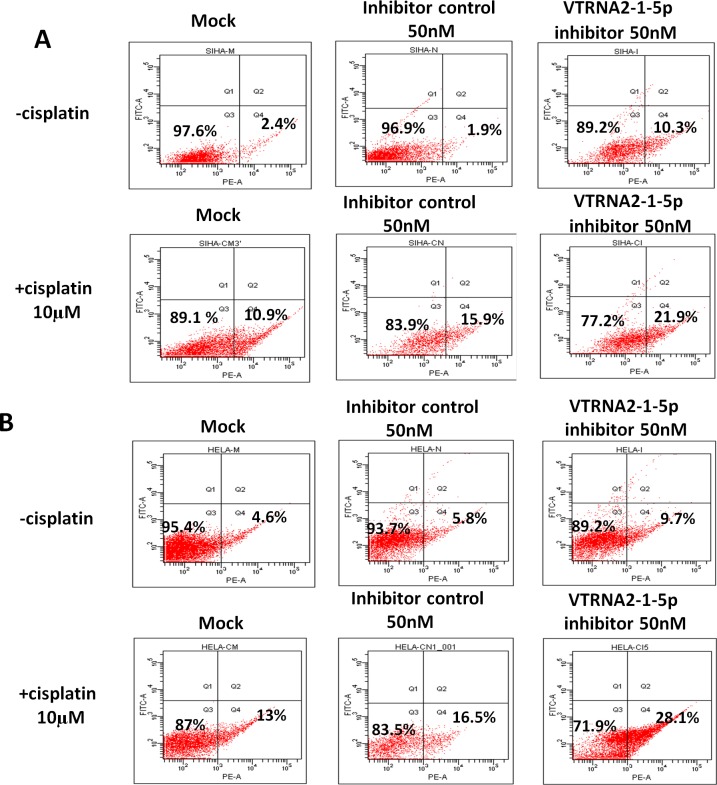
VTRNA2-1-5p inhibition promotes cancer cell apoptosis and enhances cisplatin-induced cervical cancer cell apoptosis **A.** SiHa and **B.** HeLa cells were stained with Annexin V-PE 24 hours after treatment with 50 nM VTRNA2-1-5p inhibitor, 10 μM cisplatin, or both. The image illustrates representative apoptotic cells visualized by flow cytometry.

Previous published results showed that VTRNA2-1-5p knockdown resulted in apoptosis of HeLa cells. Interestingly, we also found that knockdown induced by the exposure of cells to 50 nM VTRNA2-1-5p inhibitor increased the rate of cisplatin-induced apoptosis of cervical cancer cells (Figure 6, Supplementary Figure S5, and Supplementary Table S6). Cisplatin is a chemotherapeutic drug used in cervical cancer that can induce cancer cell apoptosis by restoring p53 functions [20, 21], and we confirmed that it induced cervical cancer cell apoptosis in a dose-dependent manner (Supplementary Figure S4). We found that approximately 22% of SiHa and 28% of HeLa cells treated with a combination of 50 nM VTRNA2-1-5p inhibitor and 10 μM cisplatin were apoptotic, and these values were significantly higher compared with those of cells treated with inhibitor only (9.7% of HeLa and 10.3% of SiHa cells) or with cisplatin only (13% of HeLa and 10.9% of SiHa cells; *p* < 0.05 in one-way ANOVA, *n* = 5, Figure 6 and Supplementary Table S6). Therefore, we concluded that exposing cervical cancer cells to the VTRNA2-1-5p inhibitor promotes apoptosis and increases the rate of cisplatin-induced apoptosis.

### VTRNA2-1-5p regulates the expression of p53 and related proteins

Lee *et al*. reported that pre-miR-886 is a specific inhibitor of RNA-activated protein kinase (PKR), which is usually considered to be apoptotic when de-repressed through the phosphorylation of eIF2α [22]. The suppression of pre-miR-886 has been shown to cause growth inhibition in HeLa and HCT116 cell lines with high nc886 expression, whereas it has not been observed to affect the proliferation of cell lines (H1299 and MDA-MB435) that are nc886 deficient [11]. We further examined the effect of VTRNA2-1-5p on the proliferation of PC-3 cells with medium nc886 expression, and the level of VTRNA2-1-5p in these PC-3 cells was eighteen-fold lower than that in HeLa cells (Supplementary Figure S6A). Interestingly, cell proliferation was reduced in PC-3 cells transfected with the inhibitor at 48 and 72 hours (*p* < 0.05), whereas exposure to the VTRNA2-1-5p mimic did not affect cell proliferation (Supplementary Figure S6C, *p* > 0.05). In addition, we found that VTRNA2-1-5p inhibitors also slightly increased apoptosis of PC-3 cells at 48 hours (apoptosis rate: 7.8% in control *vs*. 12.8% in VTRNA2-1-5p inhibitor, Supplementary Figure S6D), and this might have been caused by the PKR pathway because the VTRNA2-1-5p inhibitor also down-regulated the level of VTRNA2-1. These results provide insights into the anti-apoptotic role of VTRNA2-1-5p in HeLa and PC-3 cells. Intriguingly, the HeLa cell line expresses wild-type *p53*, whereas PC-3 is *p53*-null, raising the question of whether p53 also participates in the induction of apoptosis by VTRNA2-1-5p.

**Figure 7:**
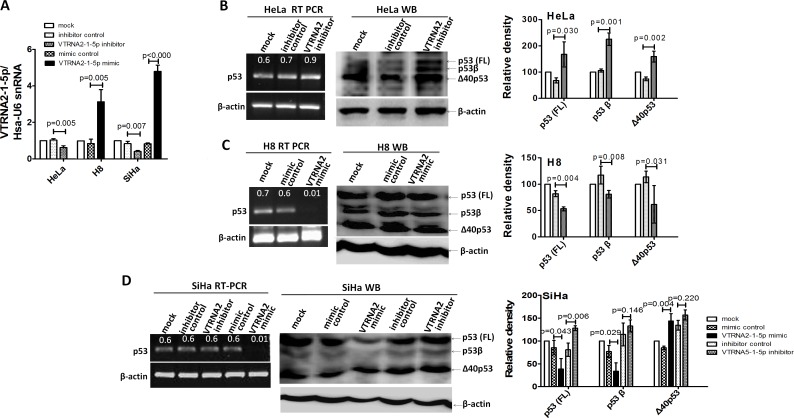
VTRNA2-1-5p affects the expression of endogenous p53 in HeLa and SiHa cells **A.** Transfection efficacy was evaluated by real-time stem-loop PCR (*n* = 3) in SiHa, H8 and HeLa cell lines transfected with the negative control or the VTRNA2-1-5p mimic or inhibitor. **B.**, **C.**, **D.** RT-PCR for mRNA and Western blotting to measure the proteins of p53 in **B.** HeLa and **C.** H8 or **D.** SiHa cells untreated or transfected with the VTRNA2-1-5p inhibitor negative control, VTRNA2-1-5p inhibitor, VTRNA2-1-5p mimic negative control or VTRNA2-1-5p mimic for 24 hours. Expression levels of p53 and its isoforms (p53β/γ and Δ40p53) were increased in the HeLa and SiHa cells because of VTRNA2-1-5p inhibition, which is consistent with the observed changes in mRNA expression. Conversely, the expression levels of p53 and its isoforms (p53β/γ and Δ40p53) were decreased in the H8 cells because of the VTRNA2-1-5p mimics, which is consistent with changes in mRNA expression. All the semi-quantitative data represent the mean±SD of three independent experiments (*n* = 3). The two-tailed Student’s t-test was used to compare the experimental values with that of the controls.

We examined the mRNA and protein expression levels of p53 during knockdown or overexpression of VTRNA2-1-5p in three cervical cell lines (*n* = 3, *p* < 0.05, Figure 7A). The RT-PCR results showed that *p53* expression was up-regulated in VTRNA2-1-5p inhibitor-transfected HeLa cells and down-regulated in VTRNA2-1-5p-mimic transfected H8 and SiHa cells (Figure 7B left). The Western blotting results showed that the level of p53 protein was significantly higher in the VTRNA2-1-5p inhibitor-transfected cells than in the corresponding negative controls (*p* = 0.03 for HeLa and *p* = 0.006 for SiHa cells, *n* = 3; Figure 7B and 7C). The expression levels of the p53 isoforms (p53β or p53γ; Δ40p53α) were also elevated in VTRNA2-1-5p-inhibitor transfected HeLa cells, whereas only p53 was elevated in VTRNA2-1-5p-inhibitor transfected SiHa cells (Figure 7B and 7C). Conversely, the levels of p53 and the p53β isoform were significantly lower in the VTRNA2-1-5p mimic-transfected cells than in their negative controls (*p* = 0.004, *p* = 0.008 for H8 and *p* = 0.043, *p* = 0.029 for SiHa cells, *n* = 3; Figure 7C). The levels of the isoform Δ40p53α were also lower in the H8 cells transfected with the VTRNA2-1-5p mimic, whereas the expression of this isoform was elevated in SiHa cells transfected with the VTRNA2-1-5p mimic (Figure 7C).

We also assessed a number of p53 pathway genes, such as *p21^WAF1^ (p21)*, *p14^ARF^ (p14)*, *MDM2, Bax* and *BcL-2*, by qRT-PCR, and these genes were searched on the KEGG database (the primers are shown in Supplementary Table S4). Our results showed that *p53, p21*, *MDM2, Bax and BcL-2* (but not *p14*) were up-regulated by 2.8-, 3.8-, 2.0-, 2.2-, and 1.7-fold, respectively, subsequent to a 2-fold down-regulation of VTRNA2-1-5p in VTRNA2-1-5p inhibitor-transfected HeLa cells; whereas *p53, p21*, *p14, Bax and BcL-2* (but not *MDM2*) were up-regulated by 2.9-, 2.1-, 1.9-, 2.5-, and 2.1-fold, respectively, subsequent to a 2-fold down-regulation of VTRNA2-1-5p in VTRNA2-1-5p inhibitor-transfected SiHa cells (*n* = 3, *p* < 0.05; Supplementary Figure S7A). These results were verified by Western blotting (Supplementary Figure S7B). A 90-kDa band corresponding to MDM2 was not observed in VTRNA2-1-5p inhibitor-transfected HeLa or SiHa cells. In the HeLa cells, p21^WAF1^ expression was found to be up-regulated, with a corresponding increase in p53. In the SiHa cells, a decrease in the smaller 14-kDa protein band, which presumably corresponded to p21^WAF1^, was observed, which is inconsistent with the observed change in its mRNA level. We speculate that the p21 level in SiHa cells was regulated by post-translation modification. Transfection with the VTRNA2-1-5p inhibitor strongly up-regulated the expression of p14^ARF^ in both HeLa and SiHa cells. The level of Bax expression also increased in both cell lines after VTRNA2-1-5p inhibitor treatment. Bcl-2 was only detected in SiHa cells, and its level of expression was not significantly changed by transfection of the cells with the VTRNA2-1-5p inhibitor; again, this result was not consistent with the observed changes in its mRNA levels. Based on these findings, we speculate that VTRNA2-1-5p down-regulates p53 expression and partly interferes with the function of the p53 signaling pathway.

### VTRNA2-1-5p directly targets p53

**Figure 8:**
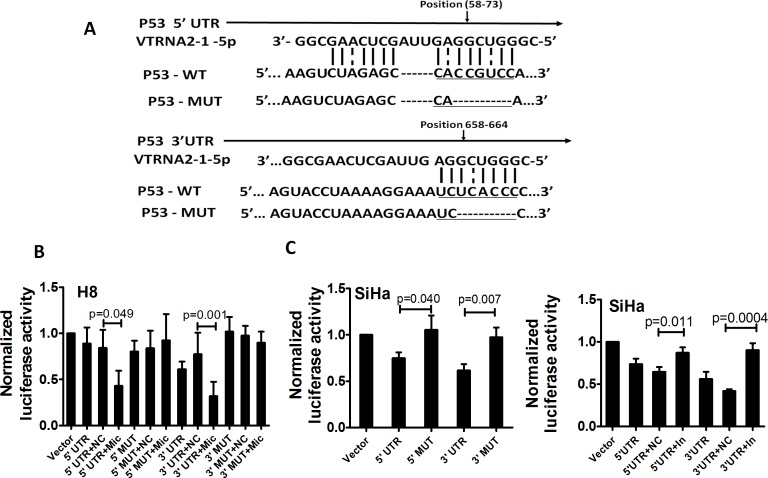
p53 is a direct target of VTRNA2-1-5p in cervical cancer cells **A.** Sequences of the VTRNA2-1-5p binding sites within the human p53 5′-UTR or 3′-UTR and schematic diagram of the reporter constructs showing the entire p53 5′-UTR or 3′-UTR sequences (p53-WT) and the mutated p53 5′-UTR or 3′-UTR sequences (p53-MUT; substitutions in the VTRNA2-1-5p binding sites are underlined). **B.** Luciferase activity in H8 cells after transfection with the p53-WT (5′-UTR or 3′-UTR) reporter or the p53-MUT (5′-UTR or 3′-UTR) reporter and in the presence of 40 nmol/L VTRNA2-1-5p mimic negative control or mimic. **C.** Luciferase activity in SiHa cells after transfection with p53-WT (5′-UTR or 3′-UTR) reporter or p53-MUT (5′-UTR or 3′-UTR) reporter (left) and in the presence of 40 nmol/L VTRNA2-1-5p inhibitor negative control or inhibitor (right). The data represent the mean±SD (*n* = 6). The two-tailed Student’s t-test was used to compare the experimental values with those obtained from the mimic or inhibitor negative controls.

We adopted the Needleman-Wunsch algorithm for global alignments to investigate whether VTRNA2-1-5p directly targets the untranslated regions (UTRs) of p53 (Figure 8A). We found that both the 5′- and 3′-UTR of p53 possessed complementary sequences to the “seed” sequence, although the matches were not perfect. Based on this finding, H8 cells (a cell line with lower endogenous VTRNA2-1-5p expression) were co-transfected with 100 ng of various p53 UTR reporters and either 40 nM of the VTRNA2-1-5p mimic or a negative control. Luciferase activity was reduced by approximately 2-fold in both UTRs after co-transfection of the cells with the VTRNA2-1-5p mimic (0.43±0.16 in the 5′-UTR and 0.32±0.16 in the 3′-UTR compared with 0.84±0.20 in the 5′-UTR and 0.77±0.23 in the 3′-UTR in the negative controls; *p* = 0.049 and *p* = 0.001, *n* = 6; Figure 7B). In addition, we measured the luciferase activity in SiHa cells (a cell line with higher endogenous VTRNA2-1-5p expression) upon transfection with various p53 UTR reporters. The luciferase activities in the p53 5′-UTR-WT and p53 3′-UTR-WT reporter groups were 0.75±0.06 and 0.62±0.07, respectively, which were significantly lower relative to the corresponding 5′-UTR-MUT and 3′-UTR-MUT controls (1.05±0.16 and 0.97±0.105, respectively; *p* = 0.04 and *p* = 0.007, *n* = 6, Figure 7C, left) because of the presence of endogenous VTRNA2-1-5p. We also co-transfected cells with 40 nM of the VTRNA2-1-5p inhibitor and 100 ng of the various p53 UTR reporters and observed that the luciferase activity of both the p53 5′-UTR-WT reporter (0.87±0.06) and the p53 3′-UTR-WT reporter (0.90±0.08) was significantly up-regulated by VTRNA2-1-5p inhibition relative to that of the negative controls (0.65±0.06 and 0.42±0.02, respectively; *p* = 0.001 and *p* < 0.0001, *n* = 6; Figure 7C, right). Overall, these results suggest that VTRNA2-1-5p directly targets the transcript of p53 by interacting with its 5′-UTR and 3′-UTR.

**Figure 9:**
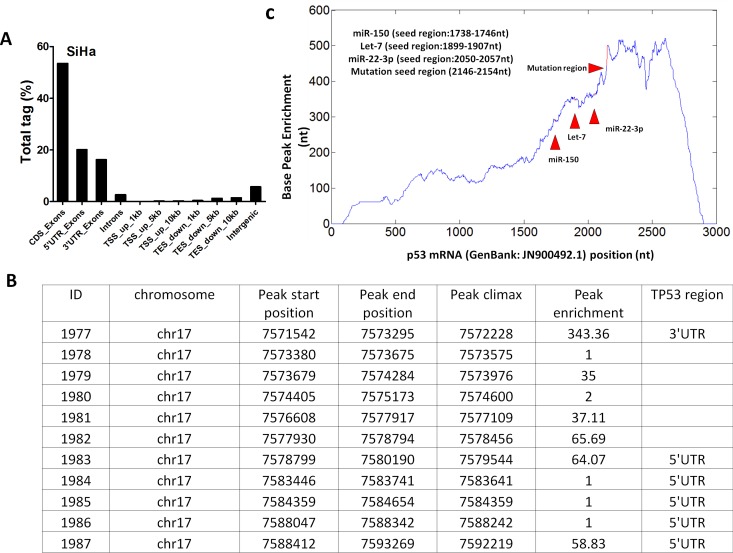
Analysis of the single nucleotide-resolution peak enriched for p53 mRNA RNAs from AGO2-associated RNP were sequenced, and the obtained RNA fragment sequences were mapped to the *p53* mRNA sequence (GenBank: JN900492.1). **A.** Regional distribution analysis of RNA fragments from anti-AGO2 RNP complexes. **B.** Peak enrichment in various regions of *p53* mRNA. **C.** The peak located between nt1738 and nt1746 of p53 mRNA is a potential miR-150 binding site, and the peak located between nt1899 and nt1907 is the potential binding site of let-7. The peak between nt2050 and nt057 is a potential miR-22-3p binding site. The peak between nt2146 and nt2154 is a potential VTRNA2-1-5p binding site within the 3′-UTR of *p53* mRNA. TSS = transcription start site; TES = transcription termination site.

To determine whether VTRNA2-1-5p directly targets p53 through the RISC mechanism, we sequenced the AGO-associated RNAs by NGS sequencing. As expected, genome mapping showed that the 5′-UTR and 3′-UTR accounted for 20.12% and 16.22% of the total non-coding region of the gene, respectively (Figure 9A). We also investigated the distribution of the AGO2-associated RNAs in p53 mRNA. One peak with an enrichment factor of 343.36 was mapped to the 3′-UTR, and two peaks with enrichment factors of 64.07 and 58.83 were mapped to the 5′-UTR (Figure 9B). A base-pair resolution peak analysis showed enrichment of the AGO2-associated RNA fragments in multiple regions of p53 mRNA (Figure 9C), including the region between nucleotides 1738 and 1746 (the region containing a potential binding site for the miRNA-150 seed), the region between nucleotides 1899 and 1907 (the potential binding site for let-7), the region between nucleotides 2050 and 2057, and the potential binding site for miRNA-22-3p. In addition, the potential VTRNA2-1-5p binding site (the region between nucleotides 2146 and 2154 in p53 mRNA) was also highly enriched in the AGO-associated RNAs. The above results indicate that VTRNA2-1-5p directly targets the UTR region of p53 mRNA.

## DISCUSSION

In this work, we were unable to rule out the presence of VTRNA2-1-5p in cervical tissue because we captured its sequence with a stem-loop structure from stem-loop qPCR products through sequencing. Mature miR-886-3p has been detected by Northern blotting in HS5, a human stromal cell line [23], and mature forms from VRNA2-1 are suggested to display tissue specificity. Moreover, we cannot rule out an miRNA-like functional role for VRNA2-1 in cervical cancer because our experiments showed that the expression levels of the most predicted targets of VTRNA2-1-5p were negatively correlated with the expression of VTRNA2-1-5p. Vault RNAs have been reported to be involved in resistance to chemotherapeutic drugs and are known to produce microRNAs through a Dicer-dependent mechanism; these microRNAs then operate in a similar manner to regular miRNA [24]. The small RNA from VTRNA1-1 (hvg-1) binds an AGO protein and down-regulates the expression of CYP3A4, an enzyme involved in drug metabolism [25]. Interactions between RNA derived from VTRNA1-2 (hvg-2) and three anti-tumor drugs have also been reported [26]. Consistent with these reports, we found that VTRNA2-1-5p was enriched in the anti-AGO2 immunoprecipitation complex, which indicates that VTRNA2-1-5p is processed by the RISC mechanism. However, we only found a minute amount of VTRNA2-1-5p in cervical tissues and cells that was too small for detection by Northern blotting. Hence, we used stem-loop qPCR to quantify the expression of VTRNA2-1-5p.

A series of reports have shown that nc886 is a tumor-suppressing ncRNA in human esophageal squamous cell carcinoma (ESCC) [27], gastric cancer [22], cholangiocarcinoma [28] and other cancers. In acute myeloid leukemia (AML) and lung cancer, hypermethylation of the nc886 promoter results in abolished expression, which eliminates the inhibitory effect [29, 30]. The genomic locus of nc886 on human chromosome 5q31 is frequently deleted in leukemia [31, 32]. Tumor surveillance by nc886 is based on its ability to bind and repress the double-stranded RNA (dsRNA)-dependent PKR. Activation of PKR by dsRNA induces the expression of proapoptotic genes of the tumor necrosis factor receptor family, including Fas and proapoptotic Bax [33]. PKR exhibits affinities for dsRNAs of various sizes, and molecules shorter than 30 bp fail to bind stably [34]. Until now, the best-characterized role of nc886 has been its binding to PKR and inhibition of PKR activity. Our data show that VTRNA2-1-5p inhibition provoked apoptosis in HeLa, a cell line with high endogenous nc886 expression, and in PC-3, a cell line with low endogenous nc886 expression. However, Lee *et al*. reported that nc886 inhibition did not affect proliferation in two nc886-deficient cell lines, H1299 and MDA-MB435. Clearly, nc886 is a critical anti-apoptosis factor in cancer cells. The apoptosis induced by VTRNA2-1-5p inhibition in HeLa and PC-3 cells was likely partly induced through the PKR mechanism because VTRNA2-1-5p inhibition also decreased VTRNA2-1 levels. Our hypothesis is that VTRNA2-1-5p is the mature form of nc886. Obviously, VTRNA2-1-5p cannot bind PKR because of its single-stranded nature and size. However, the role of VTRNA2-1-5p in apoptosis and proliferation remains unclear. Our data show that VTRNA2-1-5p inhibition promoted apoptosis in *p53-*wild HeLa more effectively than in *p53-*null PC-3 cells; thus, we cannot rule out the possibility that VTRNA2-1-5p might also interact directly with other molecules, such as p53, which is regarded as a classic pro-apoptotic molecule. In addition, VTRNA2-1-5p overexpression, which did not impact the expression of VTRNA2-1, promoted the growth of SiHa and HeLa by up-regulating p53 levels, whereas VTRNA2-1-5p overexpression did not affect the growth of *p53-*null PC3 cells.

A number of studies have demonstrated the importance of the p53 signaling pathway. The *p53* gene is often mutated in human cancers, and this mutation is found in approximately 23% of all breast cancer samples, where it is the second most frequently mutated gene [35]. Hence, mutant *p53* is believed to be the driving oncogene in breast cancer [36]. HPV-positive cervical carcinoma cells usually possess the wild-type *p53* gene without mutations, whereas the p53 protein is inactivated in these cells [37]. Our data showed strong p53 staining in breast cancer tissue and weak staining in cervical cancer tissue, a finding that is consistent with the literature [38]. Down-regulation of p53 levels is thought to be a key mechanism in cervical cancer carcinogenesis. HPV has been identified as an etiological agent involved in the pathogenesis of most cervical cancers. The so-called high-risk HPV types lead to the expression of E6 oncoproteins that bind to p53 and promote p53 degradation through an ubiquitin-dependent proteolysis system [37]. In this work, we found that VTRNA2-1-5p was highly expressed in cervical cancer cells; however, studies have indicated that most malignancies, including those of MDA-MB-231 (breast), HeLa (cervix), and HCT116 (colon) cell lines, lack pre-miR-886 [11]. We also observed VTRNA2-1-5p expression in breast cancer tissues, whereas a significant difference in VTRNA2-1-5p expression was not observed in normal para-cancerous tissues and cancerous tissues. These findings demonstrate that VTRNA2-1-5p has a specific biological role in cervical cancers. *In vitro* experiments, VTRNA2-1-5p was shown to be involved in the down-regulation of *p53* mRNA and protein levels for various p53 isoforms. To date, 12 distinct isoforms of p53 have been reported, and they are produced through alternative initiation of translation and alternative splicing [39, 40]. Isoforms of p53, p53β, p53γ, Δ133p53α, Δ133p53β, and Δ133p53γ have been detected in certain tumors and normal tissue [41]. The levels of a number of the wild-type p53 isoforms, such as FLp53, p53β, p53γ, and Δ40p53, were found to be low in HeLa, SiHa, and H8 cells because of the presence of the E6-ubiquitin-dependent proteolysis system. SiHa cells contain only one copy of HPV 16 DNA per cell and have higher levels of wild-type p53 than HeLa cells, which have 25 copies of HPV 18 DNA per cell [42].

We propose that VTRNA2-1-5p directly targets p53 UTRs and inhibits p53 transcription and translation. The results obtained using the luciferase report systems suggest that VTRNA2-1-5p targeted the transcript of p53 directly by interacting with its 5′-UTR and 3′-UTR. In addition, a RIP assay showed that potential VTRNA2-1-5p target sites in the 5′-UTR and 3′-UTR region of *p53* mRNA were enriched in the anti-AGO2 immunoprecipitation complex, although the enrichment of the 5′-UTR region was lower than that of the 3′-UTR region. This result might have been caused by the unique spatial structure that has been reported, i.e., the presence of a dsRNA region containing complementary sequences to the 5′-UTR and 3′-UTR of human *p53* mRNA [43]. The length of the 3′-UTR of *p53* mRNA (1206 bp), which contributes to the binding of the probe, is another possible explanation. Unfortunately, experiments that might demonstrate a direct interaction between VTRNA2-1-5p and p53 UTRs, such as RNase protection assays, are difficult to implement because of the minute amount of VTRNA2-1-5p in the cells.

We have also demonstrated that VTRNA2-1-5p down-regulated Bax [6] and p14^ARF^, showing that it affects the apoptosis of cervical cancer cells by targeting multiple molecules. It is well known that p21, p14, MDM2, Bax and BcL-2 are key downstream signaling molecules in the p53-dependent apoptotic pathway. In our study, p21^WAF1^, a downstream effector of p53, was shown to be up-regulated by p53 [44]. However, we also detected a small 14-kDa band that reacted with the p21^WAF1^ antibody, and the levels of this band decreased with increasing levels of p53 in SiHa cells. Huang et al. showed that this smaller 14-kDa band might be a cleaved form of p21^WAF1^ and suggested that its decrease could play an important role in p53-dependent apoptosis [45]. The expression of p14^ARF^ in HeLa and SiHa cells was found to be strongly up-regulated by the VTRNA2-1-5p inhibitor. Studies have indicated that p14^ARF^ may activate the p53 pathway by interacting with and inhibiting the ubiquitin ligase activity of MDM2 [46]. With the exception of p53 and Bax, the p53 downstream signals measured here did not show synchronous changes in mRNA and protein levels in two separate cell lines. Therefore, we support the hypothesis that VTRNA2-1-5p directly targets p53 and indirectly targets multiple molecules in the p53 pathway. However, it is still not clear whether changes in the expression of VTRNA2-1-5p are induced by HPV proteins. Thus, possible links between HPV proteins and VTRNA2-1-5p expression deserve further investigation.

In summary, we have identified a link between VTRNA2-1-5p and p53 in cervical cancer cells. We also found that VTRNA2-1-5p inhibition reduced cervical cancer cell growth *in vitro* and *in vivo*. These findings support the idea that VTRNA2-1-5p plays an important role in the apoptosis of cervical cancer cells and suggest that VTRNA2-1-5p may be a potential target in the treatment of cervical cancer.

## MATERIALS AND METHODS

### Ethics statement

Breast and cervical cancer specimens (KY-2014-005) were obtained from the Beijing Biobank of Clinical Resources, which is supported by the Beijing Municipal Science and Technology Commission (D131100005313009). The specimens were obtained with the informed consent and approval of the Ethical Review Board of Investigation in Human Beings at Beijing Obstetrics and Gynecology Hospital of Capital Medical University. The investigation was conducted in accordance with the ethical standards set forth in the Declaration of Helsinki and according to national and international guidelines.

All animal studies were conducted at the animal facility of Capital Medical University in accordance with national and institutional guidelines. This housing facility is a barrier housing facility, and it is maintained in keeping with the national standard “Laboratory Animal-Requirements of Environment and Housing Facilities” (GB 14925-2001). The care of laboratory animals and the animal experimental operations conformed to the guidelines set forth in “Animal Experimental Ethical Rules of Capital Medical University.”

### Isolation of cellular and tissue RNAs and Northern blot analysis

The human cervical carcinoma cell line HeLa (HPV 18+) and the CSCC cell line SiHa (HPV 16+) were purchased from the Chinese Academy of Science Cell Bank (Shanghai, China). The HPV 16-immortalized human cervical mucosal epithelial cell line H8 was purchased from the Cell Center of Peking Union Medical University (Beijing, China). This cell line is derived from normal squamous cervical epithelium [47]. The cells were cultured at 37°C in a 5% CO_2_ incubator in DMEM supplemented with 100 μg/mL streptomycin, 100 units/mL penicillin, and 10% fetal bovine serum (FBS), and they were routinely passaged in 2- to 3-day intervals. The characteristics of the three cell lines used in this work are summarized in Supplementary Table S2.

The isolation of miRNA from tissues and cell lines was performed using a mirVana™ miRNA Isolation Kit (AM1561). Briefly, the sample was first lysed in a denaturing lysis solution, and the lysate was then extracted with acidic phenol:chloroform, which removes most of the cellular components and yields a semi-pure RNA sample. The sample was further purified over a glass-fiber filter by special procedures to yield a size fraction enriched in miRNAs. The glass-fiber filter procedure used solutions formulated specifically for miRNA retention to avoid the loss of small RNAs, which typically occurs with glass-fiber filter methods.

Northern blotting was conducted using the High Sensitive MiRNA Northern Blot Assay Kit (Signosis, NB-1001). The probes for the Northern blotting analysis were purchased from WuHan Zhongzhi Biotechnologies Co. Ltd. (WuHan, China), and the sequences of the probes are listed in Supplementary Table S1. Briefly, 2 μg of each miRNA-enriched sample was run on a 15% polyacrylamide/8 M urea denaturing gel and electrophoretically transferred to a nylon membrane (Signosis, NB-1001) at 0.2 A for 1 hour. After transfer, the RNA was immobilized by Stratagene UV cross-linking and then hybridized to the probe at 42°C overnight in NB hybridization buffer (Signosis). The membranes were washed twice at 42°C in NB hybridization wash buffer and once in 1× detection wash buffer. After blocking the membrane with 15 ml of blocking buffer for 30 minutes at room temperature, streptavidin-HRP conjugate was added to the blot incubation solution and maintained for 45 minutes. The membranes were washed three times before adding substrates A and B, and they were then exposed to a Fujifilm LAS3000 Imager (Fuji, Tokyo, Japan).

### *In situ* hybridization (ISH) and tissue microarray immunohistochemistry (IHC)

A cervical squamous cell cancer tissue microarray (TMA, OD-CT-RpUtr03-003) containing 31 cases of squamous carcinoma and 31 cases of adjacent normal tissues and a breast cancer tissue microarray (TMA, OD-CT-RpBre03-004) containing 31 cases of infiltrative ductal breast carcinoma and 31 cases of adjacent normal tissues were purchased from Shanghai Outdo Biotechnology Co. Ltd. (Shanghai, China).

ISH was performed according to the manufacturer’s instructions using a miRCURY LNA™ microRNA ISH optimization kit (Exiqon, Vedbaek, Denmark). The details of this procedure are provided in Supplementary Methods 1. Single 3′-digoxigenin (DIG)-labeled miRCURY LNA detection probes (Exiqon) were used, and the sequences of these probes are listed in Supplementary Table S1. The snRNA U6 probe was used as a positive control and miR-159 was used as a negative control in all the experiments. VTRNA2-1-5p expression was assessed using an Aperio ImageScope v11.1.2.752 (Aperio Technologies), with three sites per spot. The expression levels were classified into four groups: (0) < 1% positive area; (1) 1% to 25% positive area; (2) 26% to 50% positive area; (3) 51% to 75% positive area; and (4) > 75% positive area. The intensity of staining was scored as 1, 2, 3 or 4, which represented negative, weak, moderate, and strong staining, respectively.

To perform IHC, paraffin-embedded tissue samples were cut into 4-μm-thick sections and pretreated at 65°C for 2 hours followed by deparaffinization in xylene and rehydration through decreasing grades of ethanol. Antigens were retrieved prior to the application of the primary antibody (anti-p53 antibody, #9282, CST, 1:50) overnight at 4°C. As a negative control, sections were incubated with PBS, and the slides were then incubated with a secondary antibody conjugated to horseradish peroxidase (HRP, 1:100; DAKO, Glostrup, Denmark) for 2 hours at room temperature. HRP activity was detected using the Super Vision method (SV). In each case, more than ten visual fields were observed or more than 1000 cells were counted per slide, and p53 protein expression was represented by a positive index corresponding to the number of p53-positive cells per 1000 cells.

### Cell lines and cell culture

The human cervical carcinoma cell line HeLa (HPV 18^+^) and the cervical squamous cell carcinoma cell line SiHa (HPV 16^+^) were purchased from the Chinese Academy of Science Cell Bank (shanghai, China). The HPV 16-immortalized human cervical mucosal epithelial cell H8 was purchased from the Cell Center of Peking Union Medical University (Beijing, China). This cell line is derived from normal squamous cervical epithelium [47]. Cells were cultured at 37°C in a 5% CO_2_ incubator in DMEM supplemented with 100 μg/mL streptomycin, 100 units/mL penicillin, and 10% fetal bovine serum (FBS), and were routinely passaged at 2 to 3-day intervals. The characteristics of all three cell lines are summarized in Supplementary Table S2.

### RNA isolation and quantitative real-time stem-loop qRT-PCR

Total RNA was extracted from the cervical cancer cell lines using TRIzol reagent (Invitrogen, Carlsbad, CA, USA). Each reverse transcriptase reaction contained a total of 0.5 μg RNA, 50 nM stem-loop RT primer, 1×RT buffer, 0.25 mM each of dNTPs, 3.33 U/mL MultiScribe reverse transcriptase, and 0.25 U/mL RNase inhibitor. The 10 μL reaction mixtures were incubated for 30 minutes at 16°C, 30 minutes at 42°C, and 5 minutes at 85°C and then held at 4°C. All the reverse transcriptase reactions, including no-template controls and RT minus controls, were run in duplicate. The stem-loop primers were 5′ - GTCGTATCCAGTGCAGGGTCC GAGGTATTCGCACTGGATACGACCCGCTT - 3′ (VTRNA2-1-5p) and 5′-GGAACGCTTCACGAATTTG-3′ (U6 snRNA as an internal control).

All the quantitative real-time PCR assays were performed using an MX3000P real-time RT-PCR system and a Brilliant II SYBR^®^ Green Q-PCR Master Mix kit (Stratagene, La Jolla, CA, USA), with RNA inputs normalized to the level of human U6 snRNA. The sequences of the forward and reverse primers for human VTRNA2-1-5p were 5′- CGGGTCGGAGTTAGCTCA-3′ and 5′-TGCGAATACCTCGGACCCTG-3′, respectively, and those for human U6 snRNA were 5′-ATTGGAACGATACAGAGAAGATT-3′ and 5′-GGAACGCTTCACGAATTTG-3′, respectively. Amplification was conducted for 10 minutes at 95°C followed by 40 cycles of 15 seconds at 95°C and 1 minute at 60°C. All the reactions were run in triplicate.

### Oligonucleotide synthesis and plasmid preparation

Chemically synthesized VTRNA2-1-5p RNA mimics and inhibitors were obtained from Invitrogen (Carlsbad, CA, USA), and their sequences, as well as those of the negative controls, are provided in Supplementary Table S3. The siRNA-expressing plasmids used to inhibit the expression of VTRNA2-1-5p were purchased from Shanghai GenePharma (Shanghai, China).

The cells were transfected with Lipofectamine 2000 reagent (Invitrogen) at approximately 70-90% confluence. Following the manufacturer’s instructions, the transfection complexes were prepared and added directly to the cervical cancer cell cultures at a final oligonucleotide concentration of 50 nmol/L. To establish stable VTRNA2-1-5p knockdown SiHa lines, SiHa cells were stably transfected with a plasmid expressing a VTRNA2-1-5p inhibitor or with a control plasmid. After transfection, SiHa cells were selected in the presence of G418.

### Colony formation and tetrazolium (MTT)-based assays

Transfected HeLa cells and SiHa cells were selected for 1 week in the presence of the antibiotic G418, and then, 1000 selected GFP-positive cells were plated in 10-cm dishes to assess the effects of transfection on their ability to form colonies after 10 days of incubation at 37°C in a 5% CO_2_ incubator. At 24, 48, and 72 hours after transfection, the cells were harvested for the MTT assays, which were performed as previously described [48]. The cell viability (% of mock) was calculated as the absorbance of NC or In/absorbance of mock × 100%. All the experiments were repeated three times.

### *In vitro* invasion assays

Cell invasion was assessed using 24-well BD Falcon™ cell culture inserts with 8-μm pores (BD Biosciences, Franklin Lakes, NJ, USA) according to the manufacturer’s protocol. Transwell membranes coated with Matrigel^®^(BD Biosciences, Franklin Lakes, NJ, USA) were used to evaluate the invasive capabilities of the transfected HeLa and SiHa cells. The cells in eight randomly selected visual fields of each membrane were counted. In all experiments, the data were collected from three chambers.

### Cell apoptosis assay

Apoptosis assays were performed on HeLa and SiHa cells 24 hours after transfection using an Annexin V-PE apoptosis detection kit (Beyotime Institute of Biotechnology, Shanghai, China) and analyzed by FACS.

### Luciferase reporter assay

The 5′-UTR and 3′-UTR of p53 and related mutant luciferase reporter plasmids were constructed based on the pmirGLO vector (Promega, San Luis Obispo, CA, USA). Mutations of the p53 5′-UTR-WT and p53 3′-UTR-WT sequences were created using a Quick-Change site-directed mutagenesis kit (Stratagene). The details of the plasmid construction technique are presented in Supplementary Materials and Methods.

For the purpose of the assay, the cells were transfected with firefly luciferase reporter vectors in 96-well plates using Lipofectamine 2000 (Invitrogen). The transfection mixtures contained 100 ng of the firefly luciferase reporter plasmid and 40 nM of a synthetic VTRNA2-1-5p mimic or inhibitor in a total volume 20 μl. The cells were collected 24 hours after transfection, and luciferase activity was measured using the Promega Dual-Luciferase™ reporter assay system. Luminescence was measured using the EnVision 2104 Multilabel Reader (PerkinElmer, Norwalk, CT, USA) with Wallac EnVision Manager software version 1.11. The cells were transfected in triplicate wells, and the experiments were repeated three times.

### Western blot analysis

The cells were lysed in RIPA lysis buffer (Sigma) with protease inhibitors, and the protein concentration of the extracts was measured using a BCA assay (Pierce, Rockford, IL, USA). The membranes were incubated overnight at 4°C with antibodies to p53 (#9282, 1:500), p14 (#2407, 1:1000), p21 (#2947, 1:800), Bax (#2774, 1:1000), and Bcl-2 (#2876, 1:800), which were obtained from Cell Signaling Technology (Beverly, MA, USA), and antibodies to MDM2 (sc-965, 1:500), which were obtained from Santa Cruz Biotechnology (Santa Cruz, CA, USA). HRP-conjugated goat anti-rabbit or anti-mouse IgG was diluted to 1:8000. The membranes were developed using an enhanced chemiluminescence reagent (GE Healthcare, Little Chalfont, Buckinghamshire, UK) and then exposed to a Fujifilm LAS3000 Imager (Fuji, Tokyo, Japan). The Western blots were evaluated by densitometry using ImageJ software (NIH, Bethesda, MD, USA). The protein amounts were normalized to that of β-actin (#4967, Cell Signaling Technology; Beverly, MA, USA).

### Cervical tumor xenograft model

Six-week-old female nude mice (BALB/c-nude) (animal ethics number 2012-X-109) were used to investigate tumorigenicity. Stable VTRNA2-1-5p knockdown or control vector SiHa cells were grown, and mice were s.c. inoculated in the dorsal flank with 6×10^6^ cells (five animals for the mock, five for the control vector, and five for the VTRNA2-1-5p inhibitor). The tumor size was measured each week, and the tumor volume was calculated as follows:

Tumor volume (mm^3^) = Length×(width)^2^/2 [49].

For end-point experiments, the animals were perfused with 4% paraformaldehyde under deep anesthesia and sacrificed two weeks after tumor cell injection, and the tumors were then removed and weighed. The neoplastic nature of the tumors was confirmed by histological examination.

### Statistical analysis

Each experiment was performed at least three times. Student’s *t*-test (two-tailed) was used to compare outcomes. The data shown in the figures represent the mean±SD. ISH data were analyzed using SPSS 19.0 software for Windows (SPSS Inc., Chicago, IL, USA). Because of the magnitude and range of the relative miRNA expression levels observed in the experiments, nonparametric tests were performed. All the tests were two-tailed, and *p* < 0.05 was considered statistically significant.

## SUPPLEMENTARY MATERIAL FIGURES AND TABLES


