# Supplementary material for: Regulation of p53 expression and apoptosis by vault RNA2-1-5p in cervical cancer cells
Source: Oncotarget. 2015 Jul 22;6(29):28371–88. doi: 10.18632/oncotarget.4948 (PMC4695066; doi:10.18632/oncotarget.4948)
Supplement: Supplementary file 1 [file oncotarget-06-28371-s001.pdf]

Regulation of p53 expression and apoptosis by vault RNA2-1-5p in cervical cancer cells

Supplementary Materials and Methods

In Situ Hybridization (ISH)

Tissue sections were de-waxed in xylenes and rehydrated through an ethanol dilution series (100% to 25%), and they were then were digested with 5 μg/mL of proteinase K for 20 minutes at 37°C to facilitate probe penetration and exposure of miRNA species. To minimize nonspecific binding based on charge interactions, the tissues were subjected to a brief acetylation reaction [66 mmol/L HCl, 0.66% acetic anhydride (v/v) and 1.5% triethanolamine (v/v) in RNase-free water]. Subsequently, the tissue sections were prehybridized at the hybridization temperature for 30 minutes in prehybridization solution. The prehybridization solution was replaced with 200 μL of hybridization solution containing 10 pmol of the DIG -labeled LNA probe, and tissues were incubated for 90 minutes at the hybridization temperature and washed thrice for 10 minutes. The slides were then incubated with blocking solution for 15 minutes, applied with anti-DIG reagent (sheep anti-DIG-AP at 1:800 in antibody dilatants) and incubated for 60 minutes at room temperature. Freshly prepared AP substrate was then applied to the sections, and the slides were incubated for 2 hours at 30°C. Subsequently, 200 μL Nuclear Fast Red™ (nuclear counterstain) was applied for 1 minute for nuclear counter staining. Finally, the sections were dehydrated, mounted and photographed. Microscopy and imaging was performed with an Aperio ImageScope instrument. In all the experiments, image exposure and processing (brief adjustment of contrast/brightness) to detect the miRNA-ISH signal were applied equally to the entire image of sections probed with specific miRNAs and their corresponding positive (snRNA U6) or negative (miR-159) controls.

RIP-ChIP assay

All the RIP-ChIP experiments were performed according to the instructions included with the *RIP-Assay Kit* (No RN1001, MBL, Japan). Anti-AGO 2A8 mouse antibody (MABE56, Millipore) was used to pull down endogenous AGO-containing miRNP complexes and their associated mRNAs in SiHa cells (1×10^8 cells). The *RIP-ChIP Assay* includes three parts: RNP immunoprecipitation (IP), RNA isolation and analysis of target RNAs. Prior to RIP, antibody-immobilized Protein G magnetic beads were prepared. Subsequently, 100 μl of sheep anti-mouse IgG-conjugated Protein G magnetic beads (Dynal, Novex) was rinsed three times with PBS and once with RIP complete lysis buffer (MBL), including freshly added 1 mM DTT, 100 U/ml RNase OUT (Invitrogen, Carlsbad, CA, USA) and 1× complete mini EDTA free protease inhibitor cocktail (Roche, Basel, Switzerland), and the beads were then incubated with 15 μg of monoclonal anti-AGO or negative control mouse IgG (Santa Cruz Biotechnology) in PBS for at least 30 minutes. SiHa cells were rinsed twice with PBS and then lysed on ice for 10 minutes in RIP complete lysis buffer. To reduce background signal, the lysate was pre-cleared with pre-blocked Protein G beads at 4°C for 60 minutes. After preclearance, 10 μl of the lysate was removed for use in the total RNA and protein analysis. The remaining lysate proceeded to RNP-IP with either AGO-Protein G magnetic beads or IgG-Protein magnetic G beads at 4°C for 90 minutes. After the beads were washed, an aliquot of beads was removed from each sample and mixed with 2× Laemmli sample buffer for the Western blot analysis. The total RNA from cell lysates and RNAs that had RNP-immunoprecipitated with anti-AGO antibodies were extracted using the *RIP-Assay Kit*. The isolated RNA was qualified with NanoDrop (Thermo Fisher Scientific, Inc.) and characterized with a Bioanalyzer system (Agilent Technologies, Inc.). The isolated

RNA fragments at approximately 300 nt were reverse-transcribed and sequenced with an Illumina HiSeq 2500 system. The first and second strand cDNA were synthesized using a SuperScript II Reverse Transcriptase kit (Invitrogen, 18064014). The raw data were screened with data collection software (Illumina), the read alignments were performed with BWA 0.7.5A, and the peak enrichments were performed by Findpeaks 4.0. The peak enrichment analysis for AGO2-associated p53 mRNA fragments was performed with MATLAB 7.0.

Luciferase Reporter Plasmid Construction

The p53 5'-UTR or 3'-UTR or its mutant luciferase reporter plasmid was constructed based on a pmirGLO vector (Promega). The pmirGLO DNA was digested with PmeI and SalI, and the 5´-protruding ends of SalI were filled in using the Klenow fragment. Blunt-end ligation was completed overnight. The pmirGLO DNA were amplified, and the sequences of the forward and reverse primers were 5'-CTCGAGTCTAGAGTCGACTCCGGTACTGTTGGTAAAGC-3' and 5'-GCTAG CGAGCTCGTTTAAATTGCCAAGCTTGGGCTGCAG-3, respectively. p53 5'-UTR was amplified using a cDNA template from HeLa cells. The sequences of the forward and reverse primers for p53 5-'UTR were 5'-ATCTTTAAACGCTAGTCCCATGTGCTCAAGACTGG-3' and 5'-TACGTCGACCTCGAGATCTGACTGCGGCTCCTCCAT-3', respectively. The PCR products of the reconstructed pmirGLO DNA and p53 5'-UTR were digested by restriction dual enzymes, ligated and sequenced, and they were named p53-5'UTR-WT. In addition, the sequences of the forward and reverse primers for p53 3'-UTR were 5'-ATC TTTAAAGACATTCTCCACTTCTTGTT-3' and 5'-TACGTCGACGAGATCGATATAAAAATGGG-3', respectively. The pmirGLO plasmid and the p53 3'-UTR PCR products were digested by

PmeI and SalI, ligated and sequenced, and they were named p53-3’UTR-WT. Mutations of the p53-5’UTR-WT and p53-3’UTR-WT sequences were created using a Quick-Change Site-Directed Mutagenesis kit (Stratagene).’

Supplementary Table

Supplementary Table S1 Sequences of VTRNA2-1-5p probe, positive control and negative control

Probe name	5’-3’
VTRNA2-1-5p (ISH)	CCGCTTGAGCTAACTCCGACCCG
VTRNA2-1-5p (High-sensitive Northern blot probe)	TCGACCGCTTGAGCTAACTCCGACCCGGGCC
Let-7e (High-sensitive Northern blot probe)	ACTATACAACCTCCTACCTCA
Sense miR-159 (negative control, ISH)	AGAGCTCCCTTCAATCCAAA
snRNA U6 (positive control, ISH)	ATTTGCGTGTCATCCTTGCG
snRNA U6 (Northern blot probe)	ATCGTTCCAATTTTAGTATATGTGCTGCCGAAGCGAGCAC

Notice: ISH, miRCURY LNA™ detection probe, 250 pmol, 3`-DIG labeled; Northern blot, Signosis, 5`-biotin-labeled probe. The high-sensitivity Northern blot probes contain two moieties: the complementary sequence of the miRNA and a tag sequence. The tag sequence was detected by an amplifier enriched with biotin molecules.

Supplementary Table S2   Characteristics of three cervical cell lines

	HeLa	SiHa	H8
HPV type and copies	HPV 18^{+}<br>25 copies / per cell	HPV 16^{+}<br>1 copy / per cell	HPV 16^{+}<br>--
Characteristics	Cervical adenocarcinoma	Cervical squamous cell carcinoma	Normal cervical epithelial cell
p53 type	Wild type	Wild type	Wild type

Supplementary Table S3   Sequences of chemically synthesized VTRNA2-1-5p mimics and inhibitor

VTRNA2-1-5p	Sequence (5’- 3’)
Mimics	GGGUCGGAGUUAGCUCAAGCGG
	GCUUGAGCUAACUCCGACCCGUU
Mimics NC	UUCUCCGAACGUGUCACGUTT
	ACGUGACACGUUCGGAGAATT
Inhibitor	CCGCUUGAGCUAACUCCGACCC
Inhibitor NC	CAGUACUUUUGUGUAGUACAA

Supplementary Table S4 Primers used for the qRT-PCR analysis of predicted has-miR-886-5p' targets and p53 pathway genes

Symbol	ACCESSION NUMBER	Forward primers (5'-3')	Reverse primers (5'-3')	PCR fragment
**Predicted has-miR-886-5p' targets**				
EGR3	NM_004430.2	GCGACTCGGTAGTCCATTACA	GGAAGGAGCCGGAGTAAGAG	114-218, 105 bp, 1 intron
ZNF785	NM_152458.5	AGAGCTCTGCAGCTTTCAGC	CGCCACCTCTTTTTGTTTTG	108-214, 107 bp, 2 intron
DMPK	NM_001081562.1	AGTACGTGGCCGACTTCTTG	CGTCCGATCACCTTCAGAAT	123-225, 103 bp, 10 intron
LAT2	NM_032464.2	GTCAGTGGTGTTGGCATCAG	CTCCTGGTGTTGCCTCTTGT	64-167, 104 bp, 11 intron
SLC25A45	NM_001077241.1	ACATGCACATCTTCCTAGCG	TGGTTTTGTAGCCGGACTTT	104-199, 94 bp, 4 intron
**Some genes of TP53 pathway from KEGG**				
TP53	NM_000546.3	GCTTTCCACGACGGTGAC	GCTCGACGCTAGGATCTGAC	115-211, 97 bp, 10 intron
CDKN1A (p21)	NM_000389.2	AGTCAGTTCCTTGTGGAGCC	CATGGGTTCTGACGGACAT	24-131, 108 bp, 2 intron
MDM2	NM_002392.2	TGTTGTGAAAGAAGCAGTAGCA	CCTGATCCAACCAATCACCT	99-198, 100 bp, 10 intron
BCL2	NM_000633	CTGAGTACCTGAACCGGCA	GAGAAATCAAACAGAGGCCG	97-202, 106 bp, 1 intron
CDKN2A (p14)	NM_058197	CGCGTACAGATCTCTCGAAT	CAGCAGCTCCGCCACTC	86-188, 103 bp, 2 intron
BAX	NM_004324	GGGTTGTCGCCCTTTTCTAC	GGAGGAAGTCCAATGTCCAG	106-213, 108 bp, 4 intron
**Housekeeping control gene**				

ACTB	NM_001101	GCACAGAGCCTCGCCTT	GTTGTCGACGACGAGCG	16-108, 93 bp, 5 intron

Notice: Predicted has-miR-886-5p' targets come from *DIANA - microT v3.0*, PITA and *RNA (2011)*, *Lee YS et al.* The genes of the TP53 pathway come from the KEGG database of PubMed.

Supplementary Table S5 p53-positive cell percentage in three cervical cell lines by IHC

	**H8** %	**HeLa** %	**SiHa** %
Positive cell rate (%)	9.2±0.075	5.6±0.064	7.3±0.056

Note: The data are expressed as the mean±SD (n=6).

Supplementary Table S6 VTRNA2-1-5p inhibition promotes and synergistically enhances cisplatin-induced apoptosis in cervical cancer cells

Cell line	Group/drug (Apoptosis rate %)	Mock %	Inhibitor control (50 nM) %	VTRNA2-1-5p inhibitor (50 nM) %
HeLa	－cisplatin	2.1±0.7	2.9±1.1	10.9±0.3★
	＋cisplatin(10 μM)	10.9±0.1	16.1±0.4	22.7±0.6★
SiHa	－cisplatin	4.4±0.1	5.5±0.5	10.3±0.2★
	＋cisplatin(10 μM)	12.5±0.2	16.6±0.1	29.1±1.3★
PC-3	－cisplatin	5.2±0.7	8.3±0.8	11.9±1.1★

Note: Flow cytometry was performed as described in the legend to Figure 6. Data are expressed as the mean±SD (n=5). ★$p<0.05$ compared with the inhibitor control, one-way ANOVA test.

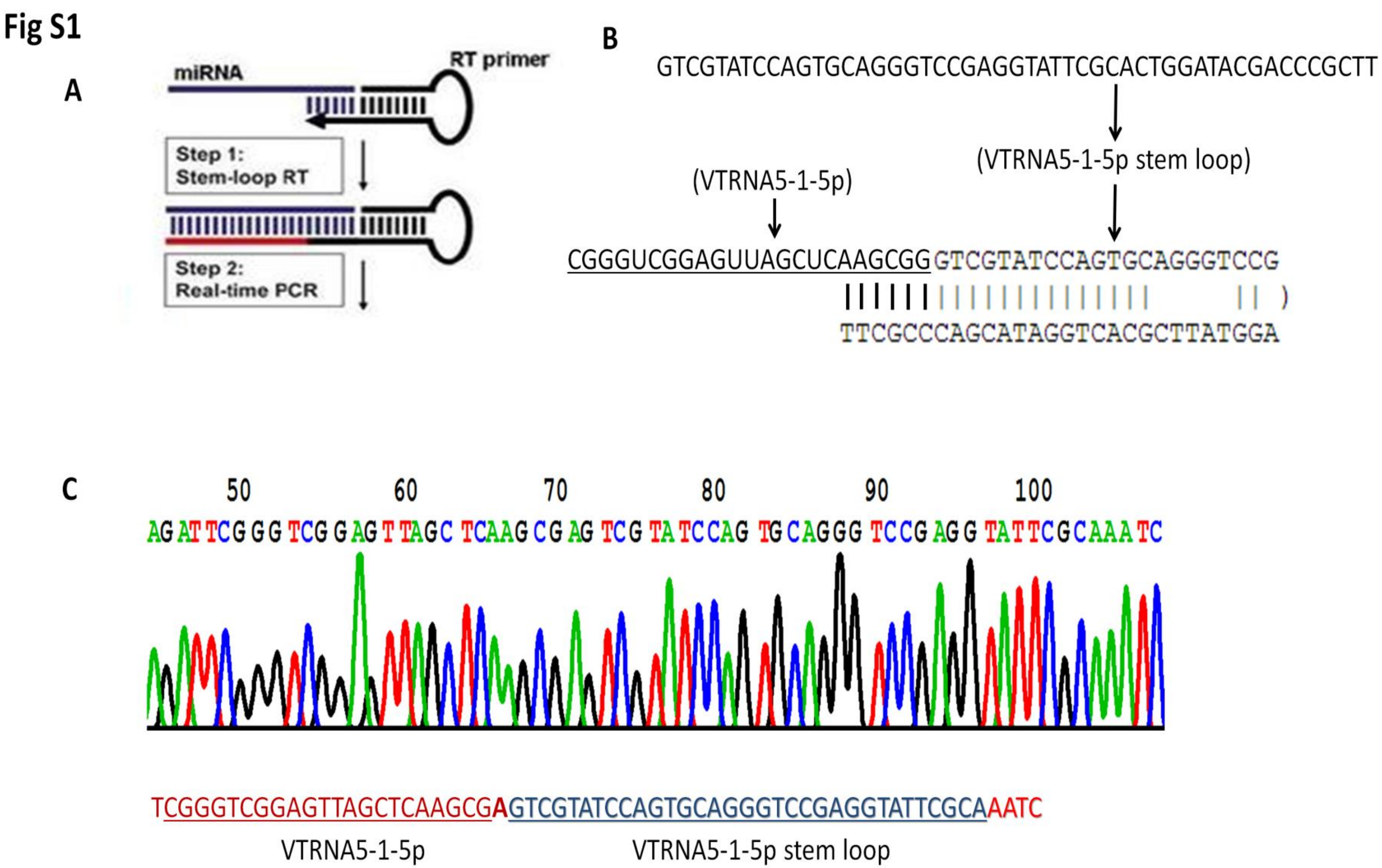

Supplementary Figure S1 Alignment results from sequencing of VTRNA2-1-5p stem-loop PCR product. (A**)** Schematic diagram of stem-loop PCR. (B) The hairpin structure of the stem-loop sequence binds with VTRNA2-1-5p. (C) The alignment results from the sequencing are shown.

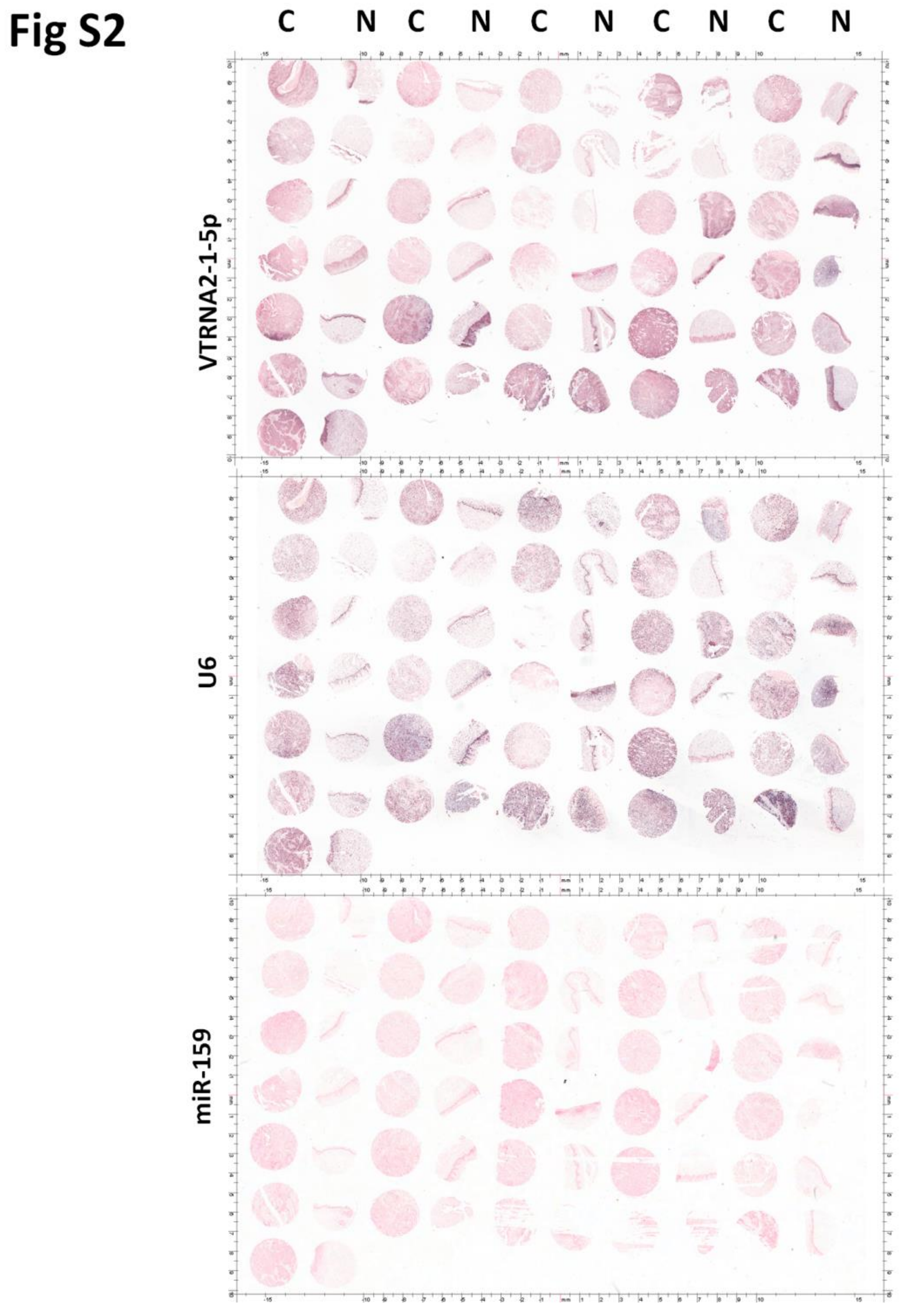

Supplementary Figure S2 VTRNA2-1-5p is overexpressed in cervical cancer tissue. Aperio image showing an ISH analysis of the expression of VTRNA2-1-5p in 31 pairs of cervical cancer tissue and matched adjacent normal tissue samples using a VTRNA2-1-5p LNA probe, positive control probe snRNA U6 or negative control probe miR-159. C, cervical cancer tissues; N, adjacent normal cervical tissues.

Fig S3

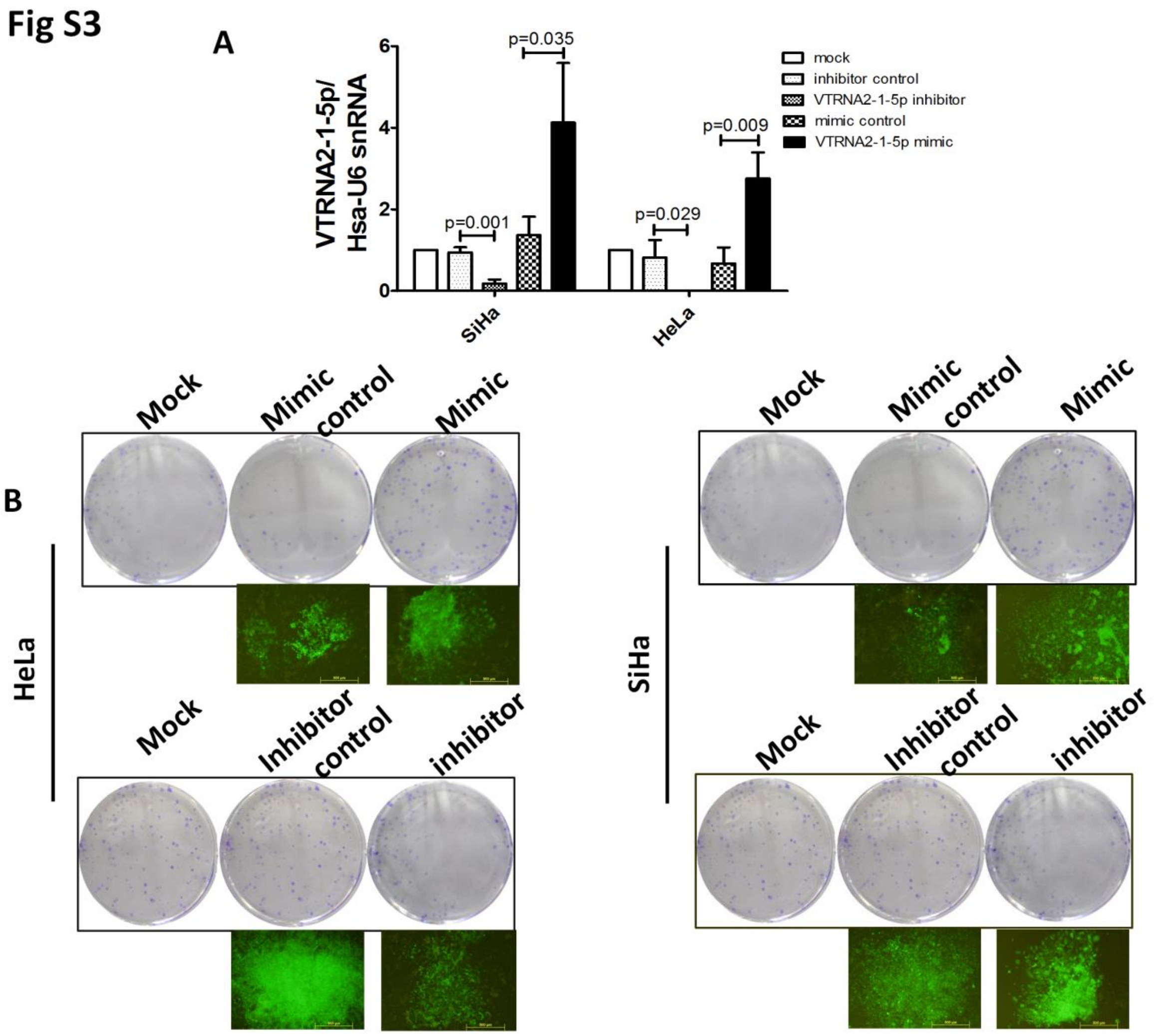

Supplementary Figure S3 VTRNA2-1-5p promotes tumor cell growth *in vitro*. (A) Transfection efficiencies of SiHa and HeLa were evaluated by stem–loop qPCR at 24 hours in Transwell experiments. (B) Photomicrographs from the colony experiment; transfection efficiency was evaluated by GFP expression.

Fig S4

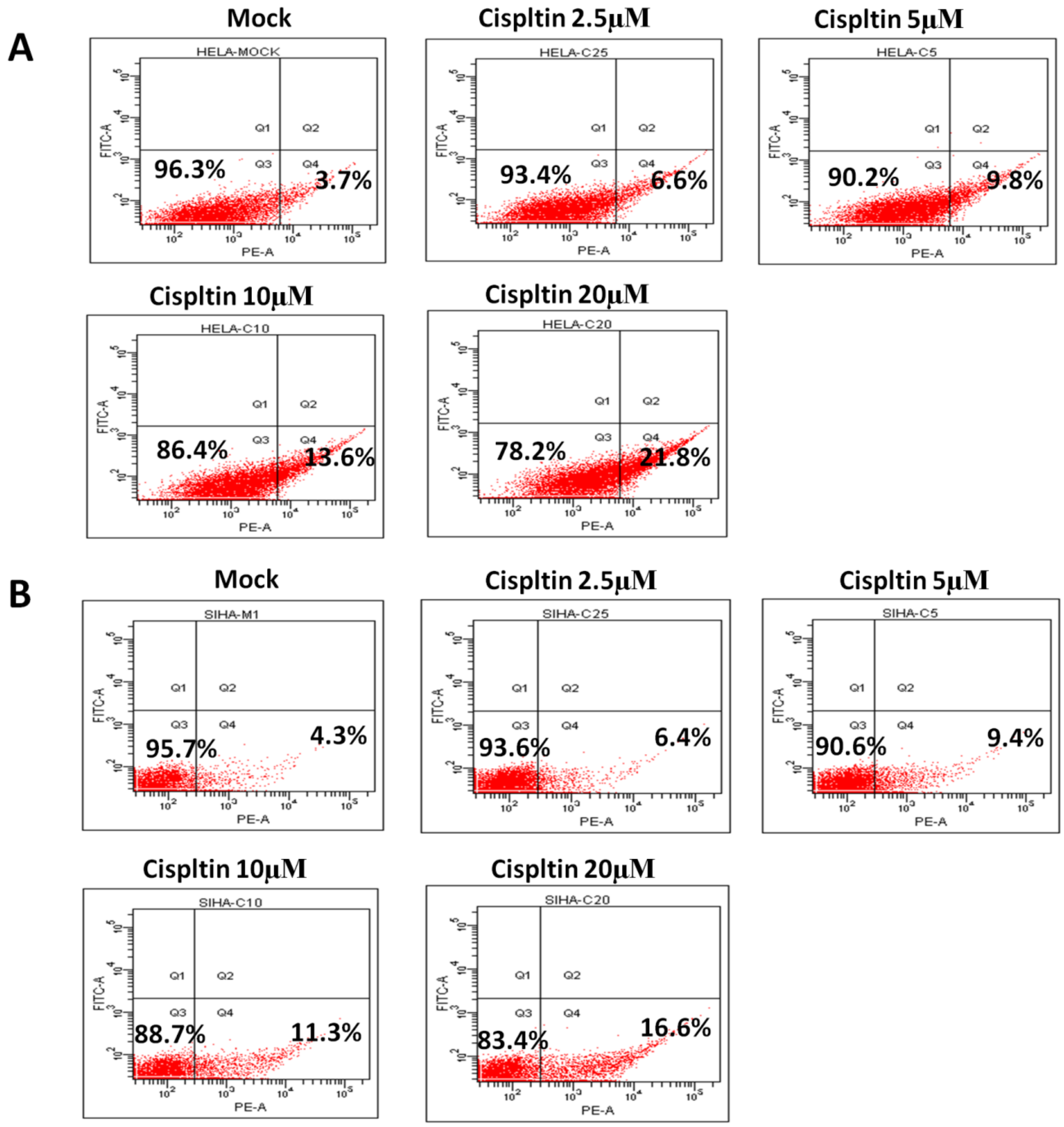

Supplementary Figure S4 Cisplatin induces cervical cancer cell apoptosis in a dosage-dependent manner. (A) HeLa, (B) SiHa cells were stained with Annexin V-PE 24 hours after treatment with 2.5, 5, 10, and 20 μM cisplatin. Photomicrographs of apoptotic cells obtained by flow cytometry are shown.

Fig S5

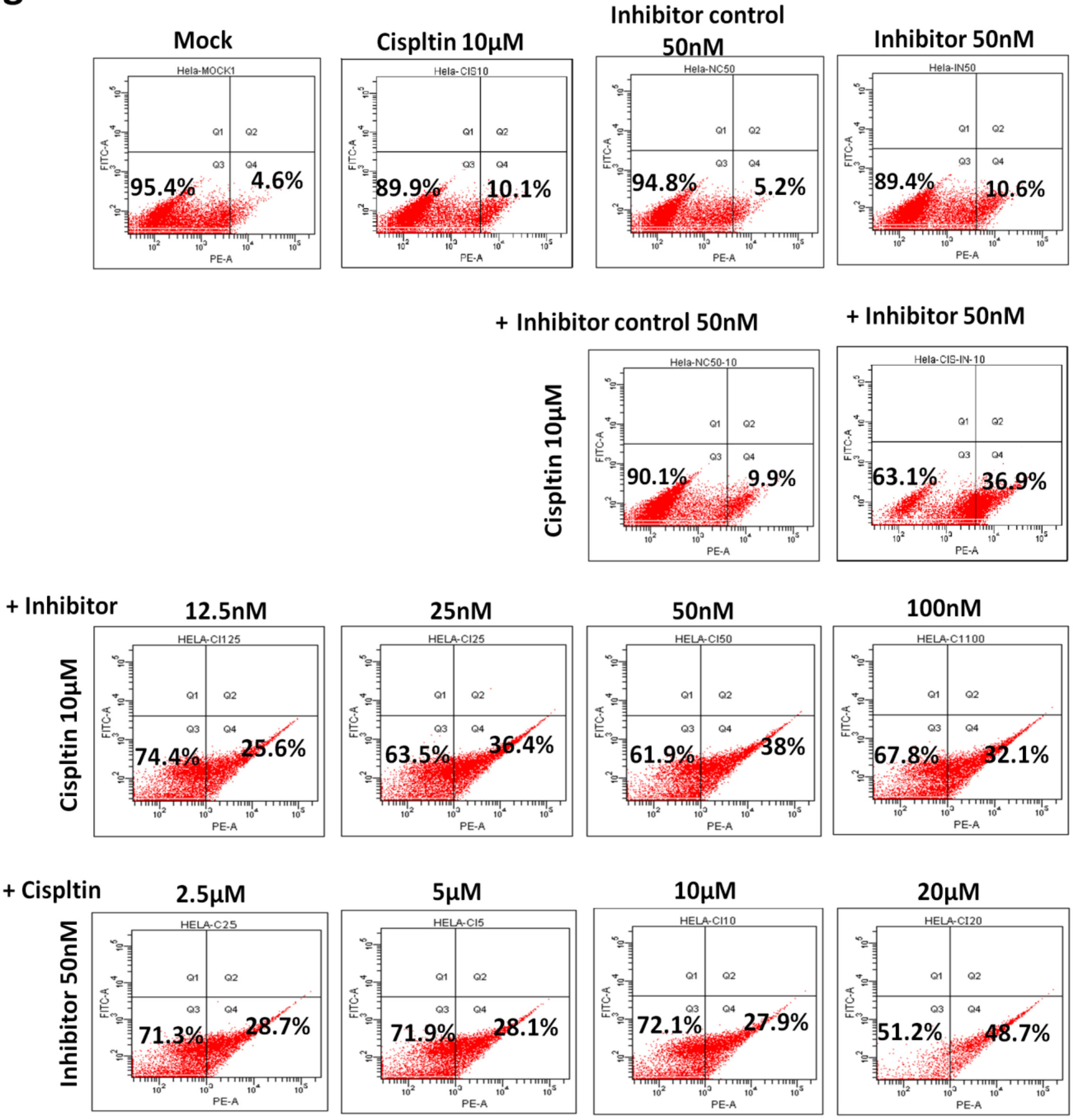

Supplementary Figure S5 VTRNA2-1-5p inhibition promotes and synergistically enhances cisplatin-induced cervical cancer cell apoptosis in a dose-dependent manner. (Upper panel) HeLa cells co-treated with 10 μM cisplatin and 50 nM VTRNA2-1-5p inhibitor or control inhibitor. (Middle panel) Cells treated with 10 μM cisplatin and then transfected with various concentrations (12.5, 25, 50, and 100 nM) of the VTRNA2-1-5p inhibitor. (Lower panel) Cells transfected with 50 nM VTRNA2-1-5p; HeLa cells were treated with various dosages (2.5, 5, 10, and 20 μM) of cisplatin.

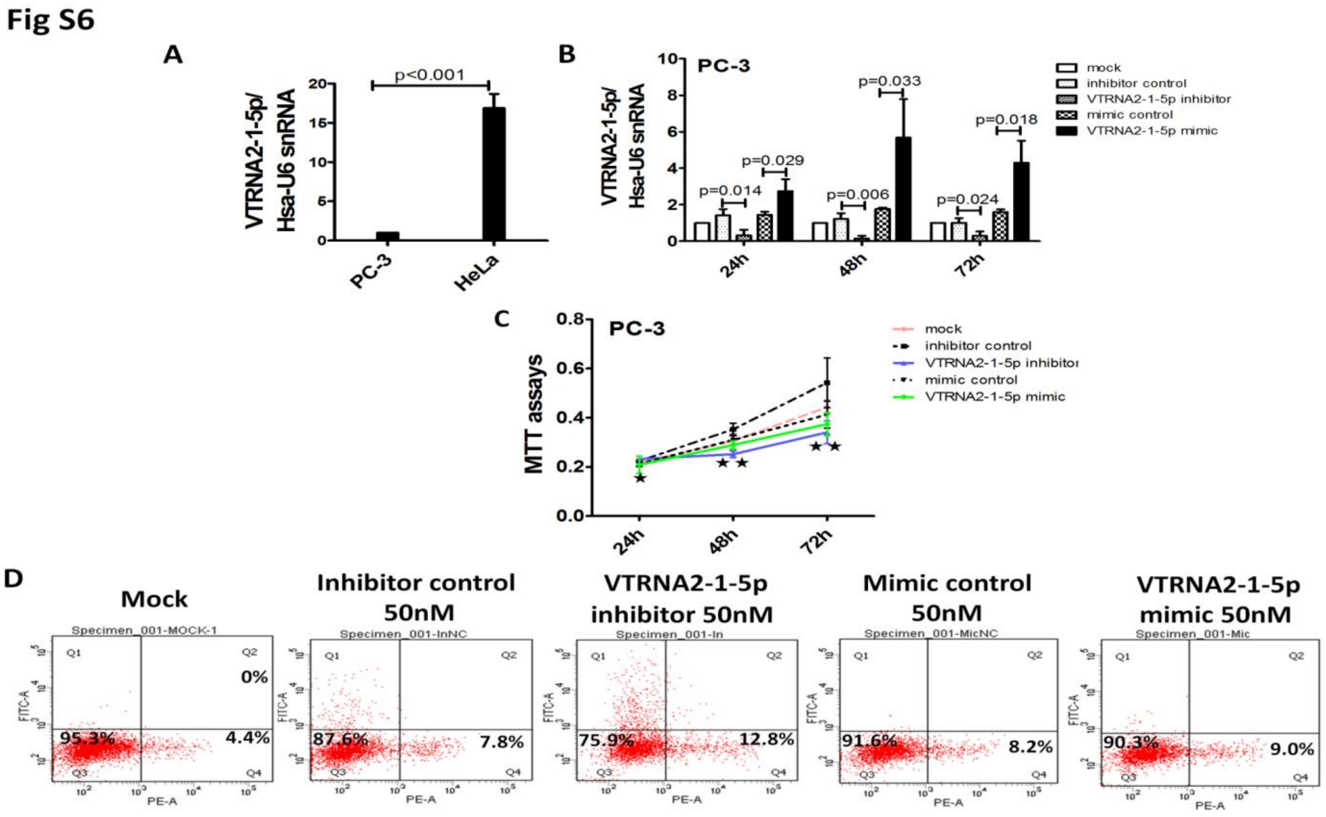

Supplementary Figure S6 VTRNA2-1-5p affects proliferation and apoptosis of PC-3 cells. (A) The expression level of VTRNA2-1-5p was compared in HeLa cells and PC-3 cells by stem-loop qRT-PCR. (B) Transfection efficacy rates of PC-3 cells 24, 48 and 72 hours after transfection with 50 nmol/L of the VTRNA2-1-5p mimic or the negative control and no transfection. (C) Down-regulation of VTRNA2-1-5p inhibits PC-3 cell proliferation at 48 and 72 hours. (D) PC-3 cells stained with Annexin V-PE 48 hours after transfection with 50 nM of the VTRNA2-1-5p inhibitor, the VTRNA2-1-5p mimic, and the control of each.

Fig S7

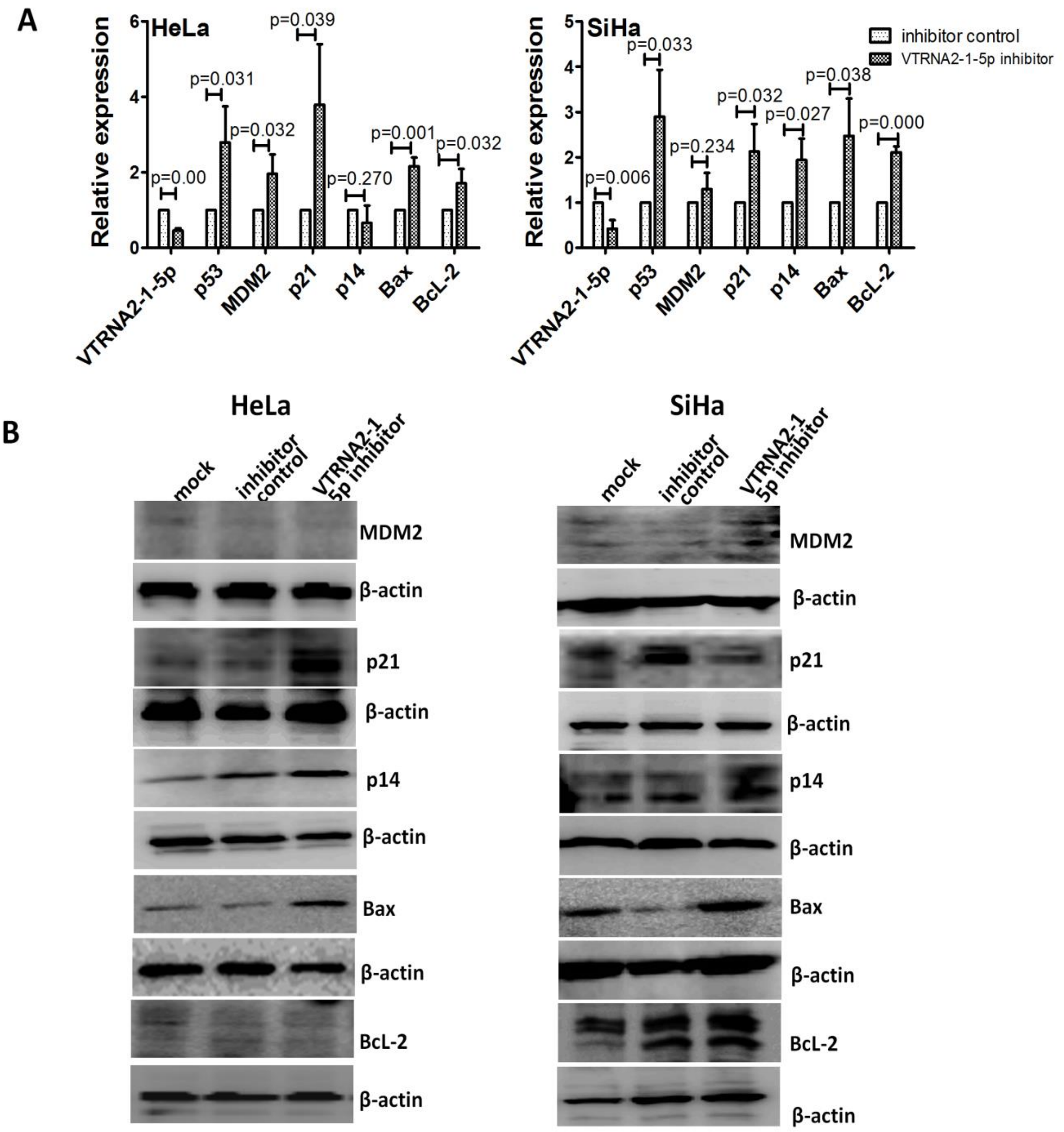

Supplementary Figure S7 VTRNA2-1-5p affects the protein expression level of endogenous p53-related factors in HeLa and SiHa cells. (A**)** The expression level of VTRNA2-1-5p was verified 48 hours after transfection by stem-loop qRT-PCT**.** The mRNA levels of p21, p14, Bax, BcL-2 and MDM2 in HeLa cells were determined using qRT-PCR 48 hours after transfection. (B) Immunoblotting of p21, p14, Bax, BcL-2 and MDM2 in HeLa and SiHa cells untreated or transfected with the VTRNA2-1-5p inhibitor negative control or inhibitor. β-actin served as a loading control.
